# Adherence to Protocol Registration Among Systematic Reviews on Photobiomodulation: A Meta‐Research Study

**DOI:** 10.1111/jep.70346

**Published:** 2026-01-08

**Authors:** Giovanna Marcilio Santos, Giullia Carvalho Mangas Lopes, Kamilla Mayr Martins Sá, Giulia Almirón da Rocha Soares, Marcela Letícia Leal Gonçalves, Sandra Kalil Bussadori, Elaine Marcílio Santos, Ana Luiza Cabrera Martimbianco

**Affiliations:** ^1^ Universidade Metropolitana de Santos Santos São Paulo Brazil; ^2^ Postgraduate Program of Health and Environment Universidade Metropolitana de Santos Santos São Paulo Brazil; ^3^ Universidade Nove de Julho (Uninove) São Paulo Brazil

**Keywords:** evidence‐based practice, low‐level light therapy, methods, photobiomodulation, systematic reviews

## Abstract

**Aims and Objectives:**

This meta‐research study mapped systematic reviews on photobiomodulation (PBM), encompassing low‐level laser therapy (LLLT) and light‐emitting diode (LED) modalities, to evaluate whether protocols were registered, whether published reviews adhered to and updated their PROSPERO records, and whether topic redundancy occurred across reviews.

**Methods:**

Comprehensive searches were conducted in MEDLINE (via PubMed) and Epistemonikos on June 2, 2025. Data were extracted on protocol registration, registry type, update status on PROSPERO, deviations between registered protocols and published reviews, topic redundancy, and the use of certainty of evidence assessment tools.

**Results:**

A total of 285 systematic reviews (with and without meta‐analysis) published between 1999 and 2025 were included, of which approximately 40% addressed dentistry and orofacial dysfunctions. Among these, 129 reviews (45%) had a registered protocol, with 125 (96.9%) on the PROSPERO platform and four (3.1%) on other registries, including INPLASY and the Open Science Framework. Despite publication, 72% (90/125) of PROSPERO records remained classified as “ongoing,” reflecting limited updates to the registry status. Deviations between registered and published protocols occurred in 22.4% (29/129) of reviews, mainly involving unplanned outcomes, modifications in search strategies, or changes in analytical methods. Redundancy was frequent, with multiple reviews addressing identical or highly similar clinical questions, such as 20 reviews on orthodontic treatment and 18 on temporomandibular dysfunction. Assessment of the certainty of the evidence was also limited, with only 18.5% (53/285) of reviews applying the GRADE approach, which was reported exclusively in reviews with meta‐analysis.

**Conclusion:**

These findings highlight low adherence to protocol registration recommendations, limited status updating, and high levels of duplication, combined with infrequent assessment of evidence certainty. Strengthening prospective registration and transparent reporting is essential to ensure that systematic reviews on PBM provide trustworthy, reproducible evidence to guide clinical decision‐making and inform healthcare policy. The study protocol is available at https://osf.io/9vknf/.

## 1 Introduction

Evidence‐based practice relies on the critical appraisal and synthesis of research evidence to inform clinical decision‐making. Within established evidence hierarchies, systematic reviews represent the highest level of methodological rigor, as they integrate findings from multiple primary studies using explicit, transparent, and reproducible methods. When rigorously conducted, they minimize bias, enhance the reliability of conclusions, and inform both clinical guidelines and health policy decisions [1, 2, 3]. The methodological rigor and transparency that underpin high‐quality systematic reviews are especially critical in rapidly evolving fields such as photobiomodulation (PBM), where the growing volume of research demands careful synthesis to support clinical decision‐making [4].

Photobiomodulation therapy (PBM), also known as low‐level light therapy (LLLT), is a noninvasive therapeutic modality that employs red and near‐infrared light, delivered by low‐power or high‐intensity lasers and light‐emitting diodes (LEDs), to induce photochemical and photobiological responses in cells and tissues [5, 6]. PBM has demonstrated a wide range of therapeutic applications across medical and dental fields, including pain management, tissue repair, and inflammation control [6, 7].

As interest in the therapeutic effects of photobiomodulation grows, the number of primary studies, and, consequently, systematic reviews, has increased markedly, resulting in redundancy within the evidence base [4]. A PubMed search conducted in September 2025 using the MeSH term ‘photobiomodulation’ retrieved more than 11,000 references, illustrating the rapid expansion of research in this field.

While PBM has been the subject of numerous reviews, the reliability of these syntheses depends on rigorous and transparent methodology [1, 2]. One key methodological step is the prospective registration of the systematic review protocol, which enhances transparency by allowing comparisons between planned and published methods. Registering protocols helps mitigate conduction and reporting bias, prevents unnecessary duplication, and reduces research waste. Additionally, it enables reviewers to document deviations from the original protocol, strengthening the integrity of the review process [2, 3].

Leading organizations, such as the Cochrane Collaboration and the Joanna Briggs Institute (JBI), require a pre‐specified protocol and publish it in their databases before initiating the review [1, 2]. In 2011, with funding from the UK National Institute for Health Research (NIHR), the UK Center for Reviews and Dissemination launched the International Prospective Register of Systematic Reviews (PROSPERO), the first international database for prospectively registered non‐Cochrane systematic reviews. While PROSPERO remains the most widely used platform, alternative options for protocol registration include the Registry of Systematic Reviews/Meta‐Analyses in Research Registry and INPLASY, as well as publication in scientific journals or preprint databases [2, 3].

Despite established guidelines recommending prospective protocol registration [1], many systematic reviews on therapeutic interventions remain unregistered or inconsistently updated, limiting the ability to assess methodological rigor and reproducibility. In the PBM field, this gap is particularly concerning; redundant or unregistered reviews can yield misleading conclusions, potentially confusing clinicians and impeding evidence‐based decisions. Strengthening transparency and prospective registration practices is therefore essential to enhance the trustworthiness of PBM evidence and to reduce research waste. Thus, this meta‐research study aimed to systematically map published systematic reviews on PBM for any health condition. The primary objective was to quantify the proportion of reviews with a registered protocol, assess adherence of published reviews to their registered protocols, and verify whether PROSPERO records were appropriately updated. The secondary objective was to identify clinical redundancy across reviews addressing similar research questions. The scientific contribution of this study was to identify gaps in transparency and prospective protocol registration practices within PBM systematic reviews, thereby supporting more credible and reproducible evidence syntheses to inform clinical and policy decision‐making.

## 2 Methods

This study followed the recommendations proposed by Murad et al. [8] for reporting meta‐research studies and the pertinent items of the Preferred Reporting Items for Systematic Reviews and Meta‐Analyses (PRISMA 2020) [9]. The study protocol was registered on the Open Science Framework and is available at https://osf.io/9vknf/.

### 2.1 Eligibility Criteria

We included systematic reviews (with or without meta‐analysis) published in peer‐reviewed journals that evaluated the use of photobiomodulation (PBM) in any form, whether delivered by LLLT or LED‐based therapies, for the treatment of health conditions across all areas of healthcare. Scoping reviews, umbrella reviews, narrative reviews, and network meta‐analyses were excluded because their methodological objectives and structures differ from those of conventional systematic reviews, which were the specific focus of this meta‐research. Systematic reviews that evaluated PBM in combination with other interventions, including experimental studies (in vivo or in vitro) were also excluded. Cochrane reviews were not considered, as protocol registration is mandatory. If updated reviews of the same review team were identified, only the first publication was considered.

### 2.2 Outcomes of Interest

- Protocol registration: Systematic review protocols registered on the PROSPERO platform (https://www.crd.york.ac.uk/prospero/) or available through other sources (e.g., scientific journals, preprint repositories, or alternative registries). Prospective registration was defined as protocols recorded before the start of the systematic review, verified by comparing the registry record date with the review's reported start date. Registry records were accessed in July 2025.
- Protocol adherence: Concordance between the methodological planning specified in the protocol and the procedures reported in the completed review, assessed across four critical domains: databases searched, eligibility criteria (PICO), risk of bias assessment methods, and planned synthesis methods.
- Review status updates: Registration of review completion or progress status on the PROSPERO platform.
- Redundancy: Extent of overlap and duplication among PBM systematic reviews, categorized by clinical condition and/or population analyzed.

### 2.3 Search Strategy for Identifying Systematic Reviews

A comprehensive search strategy was developed and conducted on June 2, 2025, to retrieve a representative sample of systematic reviews from the Medical Literature Analysis and Retrieval System Online (MEDLINE, via PubMed) and Epistemonikos, a specialized database dedicated exclusively to systematic reviews and evidence syntheses. No restrictions were applied regarding publication date or language. The complete search strategies are provided in Supporting Information S1.

### 2.4 Study Selection

Two authors (GMS and ALCM) independently screened the references obtained from the search strategies using the Rayyan platform [10] by title and abstract. Duplicates were removed by using the EndNote web software. The potentially eligible reviews were analyzed in full text to confirm inclusion or exclusion. Two additional independent authors (GCML and EMS) resolved divergences by consensus. Inter‐rater reliability between reviewers was assessed using Cohen's kappa (κ). Cohen's *κ* values were interpreted according to established levels, with values above 0.80 indicating strong agreement [11].

### 2.5 Data Extraction

Two independent authors (KMMS and MLLG) collected the data from included systematic reviews using a pre‐established data extraction form, including the following characteristics: year of publication, population analyzed (e.g., clinical condition), protocol registration platform (PROSPERO or another type of publication), adequacy between protocol and complete review regarding the research question aspects (mainly the outcomes of interest), and PROSPERO review status (review ongoing or review completed). A third author (GARS) resolved any disagreements that arose during this process.

### 2.6 Data Analysis

Data were grouped by publication year, clinical field, protocol registration status, registry platform, and adherence characteristics. Descriptive statistics were used to summarize the data, including absolute frequencies for the proportion of systematic reviews with appropriately registered protocols, the clinical area of treatment, and the review status. Additionally, RStudio (version 2025.05.1 + 513) was used to estimate and visualize the temporal trend analysis of protocol registration across publication years.

## 3 Results

**Figure 1:**
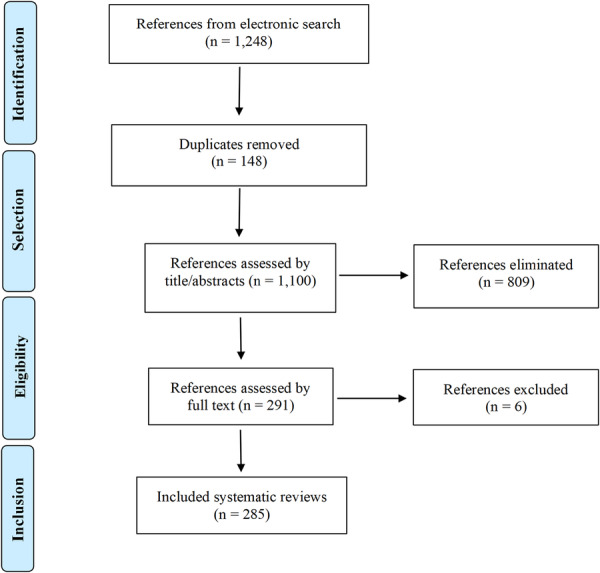
Flowchart of the study selection process.

The search strategies retrieved 1248 references. After removing 148 duplicates, 1100 records were screened in the first stage of the selection process. Of these, 809 were excluded by title and abstract because they did not meet the eligibility criteria. Subsequently, 291 systematic reviews were assessed in full text, of which six were excluded because they involved experimental primary studies (in vivo or in vitro) (Supporting Information S2). Reviewer agreement was high across all screening phases, with 95% concordance and strong inter‐rater reliability (*κ* = 0.88 for title/abstract screening and *κ* = 0.91 for full‐text screening). Finally, 285 systematic reviews met the inclusion criteria and were included in this study (Figure 1). A complete list of the references for the included systematic reviews is provided in Supporting Information S3.

### 3.1 Characteristics of the Included Studies

The 285 systematic reviews included in this study were published between 1999 and 2025. They addressed a range of health conditions, with a predominant focus on evaluating the effects of PBM on pain, inflammation, and tissue healing. Approximately 40% (111/285) of the reviews were related to dentistry and orofacial dysfunctions, followed by musculoskeletal disorders (19%, 54/285). Table 1 presents the most frequently studied clinical conditions, the time intervals in which overlapping reviews were published, and the distribution of topics. Detailed characteristics of each included systematic review are provided in Supporting Information S4.

**Table 1:** Clinical conditions assessed in the included systematic reviews.

Clinical conditions	Frequency (%)	Publication year range
Orthodontic treatment	20 (7.0)	2010 to 2025
Temporomandibular disorder	18 (6.3)	2011 to 2025
Cancer therapy‐induced oral mucositis	17 (6.0)	2011 to 2025
Dermatological conditions	13 (4.6)	2017 to 2025
Muscle performance and exercise recovery	9 (3.2)	2013 to 2025
Periodontal disease (surgical and non‐surgical)	9 (3.2)	2008 to 2021
Burning mouth syndrome	8 (2.8)	2016 to 2025
Oral lichen planus	7 (2.5)	2017 to 2025
Endodontic interventions and complications	7 (2.5)	2014 to 2024
Diabetic peripheral neuropathy/foot ulcer	7 (2.5)	1999 to 2025
Knee osteoarthritis	7 (2.5)	2007 to 2024
Carpal tunnel syndrome	7 (2.5)	2016 to 2025
Tendinopathies	6 (2.1)	2008 to 2022
Breast cancer‐related lymphedema	6 (2.1)	2012 to 2022
Dental implants	6 (2.1)	2014 to 2022
Third molar extraction	6 (2.1)	2012 to 2022
Myopia	5 (1.8)	2024 to 2025
Aphthous ulcers	5 (1.8)	2015 to 2025
Bone repair/Bone healing maxillary or palatal expansion	5 (1.8)	2017 to 2022
Neck pain and disorders	5 (1.8)	2005 to 2022
Androgenetic alopecia	5 (1.8)	2017 to 2020
Plantar fasciitis	5 (1.8)	2019 to 2025
Inferior alveolar nerve injury and preservation	5 (1.8)	2019 to 2024
Orthognathic surgery	4 (1.4)	2018 to 2022
Gingival lesions	4 (1.4)	2018 to 2022
Xerostomia	4 (1.4)	2012 to 2024
Tooth sensitivity	4 (1.4)	2017 to 2025
Lateral epicondylitis	3 (1.1)	2008 to 2014
Other conditions (< 2 systematic reviews each)	78 (27.4)	2012 to 2025

### 3.2 Systematic Review Protocol Registry

**Figure 2:**
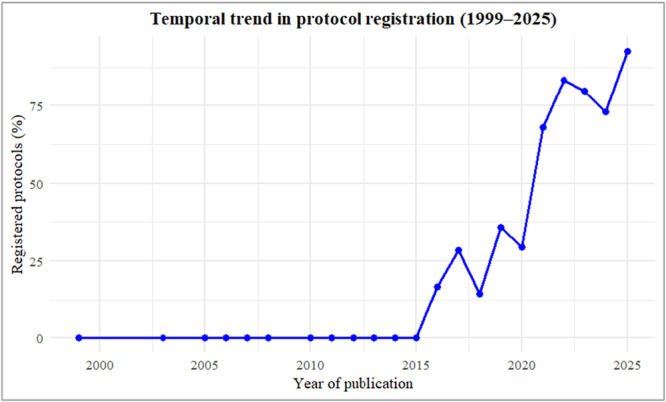
Temporal trend in protocol registration among systematic reviews on PBM (1999–2025).

Among the 285 systematic reviews included in this study, 129 (45%) had a registered protocol, all published between 2016 and 2025. Of these, 125 reviews (96.9%) were registered on the PROSPERO platform, while four reviews (3.1%) were registered on other platforms: INPLASY (*n* = 1), the Open Science Framework (OSF) (*n* = 2), and one protocol was published within the International Evidence‐Based Guideline for the Assessment and Management of Polycystic Ovary Syndrome. The temporal trend analysis (Figure 2) showed that no systematic reviews on PBM reported registered protocols before 2015. From 2016 onward, a progressive increase was observed, with fluctuations over time and a marked rise after 2018. In the most recent period (2024–2025), more than 70% of reviews reported a registered protocol, indicating substantial improvement in adherence to prospective registration practices.

**Figure 3:**
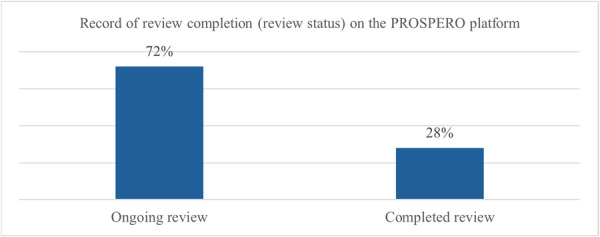
Status of PROSPERO‐registered reviews.

Even though all 125 PROSPERO‐registered reviews had already been published, the platform still listed 90 (72%) as ‘ongoing’ and 34 (27.2%) as ‘completed,’ as illustrated in Figure 3. In one review, the protocol was not found in the PROSPERO platform (0.8%).

Notably, most reviews with registered protocols were prospectively registered (88/129, 68.2%), suggesting a recent upward trend in adherence to protocol registration practices.

### 3.3 Assessment of Consistency Between Protocol Methodology and Reporting in Completed Systematic Reviews

Among the 129 systematic reviews with registered protocols, 29 (22.4%) exhibited discrepancies between the registered protocol and the final publication. Most deviations were minor, affecting one or two methodological domains, such as the addition of unplanned search databases (*n* = 14), changes in the risk‐of‐bias assessment tool (*n* = 2), or omission of the planned GRADE assessment (*n* = 4). In contrast, major deviations, more likely to influence the interpretation of review findings, were less frequent (*n* = 6, 4.6%) and primarily related to alterations in outcomes (*n* = 5), intervention or comparator characteristics (*n* = 3), or participant eligibility criteria (*n* = 1). Overall, most discrepancies reflected procedural adjustments rather than substantial methodological changes that could compromise review validity (Supporting Information S4).

## 4 Discussion

This meta‐research study found that less than half of the systematic reviews on PBM therapy had a registered protocol, despite most reporting adherence to PRISMA guidelines. This inconsistency reflects a gap between reporting transparency in PBM evidence synthesis. Since the publication of PRISMA (2009) and the launch of the PROSPERO platform (2011), prospective protocol registration has become an essential safeguard against selective outcome reporting, redundancy, and low reproducibility in systematic reviews [2, 9]. However, some barriers may explain the low adherence to this methodological step: many researchers have primarily clinical rather than methodological training, few journals explicitly require registration verification, and the International Committee of Medical Journal Editors (ICMJE) does not mandate registration for systematic reviews [12]. The absence of institutional and editorial oversight, combined with limited knowledge of methodological guidelines, likely contributes to the underutilization of protocol registries.

Although the number of registered PBM systematic reviews has increased over time, particularly after 2018, as demonstrated by our current temporal trend analysis, stronger educational initiatives and structural incentives from institutions, funders, and journals are still required to make protocol registration a standard step in the review process.

These findings are consistent with evidence from other health research fields. A meta‐research study found that only 21% of systematic reviews in ten general and internal medical high‐impact journals had registered or published protocols, and among reviews claiming adherence to PRISMA, only 22% had actually registered a protocol [13]. This reinforces that the problem is not limited to PBM but reflects a broader culture of limited methodological transparency. As highlighted by both our analysis and previous studies, there remains a need to raise awareness among authors about the importance of prospective protocol registration to enhance transparency, research quality, and, consequently, informed clinical decisions [14, 15].

Nevertheless, registration alone is not sufficient. The research transparency depends on maintaining and updating the information recorded in registries. In our analysis, although nearly all registered PBM protocols were hosted in PROSPERO, 72% remained classified as ‘in progress’ even after the corresponding reviews had been published. This lack of status updates compromises the traceability of review development and reduces the practical value of registries as tools for methodological planning and monitoring [16].

The findings of this meta‐research study also indicate that approximately one in four registered PBM reviews presented inconsistencies between the protocol and the final publication. Most discrepancies were minor, such as the inclusion of additional databases or the replacement of the risk of bias assessment tool, and may reflect reasonable methodological refinements made during the review process. However, a smaller subset of reviews reported significant changes, including unplanned outcomes or alterations in eligibility criteria. These major inconsistencies can introduce selective reporting bias, particularly through the omission of negative or non‐significant findings. Consequently, the clinical efficacy of PBM may be overestimated and the certainty of the evidence misinterpreted, leading health professionals to overvalue the therapeutic benefits and potentially make suboptimal treatment decisions [3, 16].

Still regarding methodological aspects, an additional finding of this study concerns the assessment of the certainty of the body of evidence using frameworks such as the GRADE approach. Although not a predefined objective, we explored this aspect a posteriori to better understand current practices among PBM reviewers. Among the 129 registered protocols, only 8% explicitly stated their intention to make this certainty assessment, and three of them did not implement it in the published review. Overall, only 18.5% of the included systematic reviews assessed the certainty of the evidence, a critical methodological step for interpreting results and informing clinical and policy decisions. It is worth noting that, following the recent update to the PROSPERO platform in 2025, a specific field was added for authors to report their plans for assessing the certainty of the evidence, a feature that was not available when most of the reviews included in this study were registered or published. This improvement may facilitate greater consistency and transparency in future systematic reviews.

The methodological challenges observed in this sample of systematic reviews on PBM reflect those found in other biomedical fields but are amplified by the diversity and rapid expansion of PBM research. PBM encompasses multiple health domains, including dentistry, rehabilitation, dermatology, and oncology, each with distinct terminologies, outcome measures, and intervention protocols. This heterogeneity precludes standardization and increases the likelihood of redundant or overlapping reviews. In this study, for example, 20 reviews addressed orthodontic treatment and 18, temporomandibular dysfunction, often with similar objectives and inclusion criteria. Such redundancy wastes research resources and can lead to conflicting conclusions, confuse clinicians, and difficult the translation of PBM evidence into clinical practice [2]. The high volume of overlapping reviews suggests that researchers may not consult existing registries before initiating new projects, reinforcing the importance of prospective registries as preventive and coordinating mechanisms.

Additionally, peer review and editorial oversight could play a decisive role in improving protocol compliance. Strengthening editorial policies could address this gap. For example, journals could mandate inclusion of a registry link, require explanation of any deviations from the original protocol, or perform automated checks comparing registered and published methods. Such practices would align PBM journals with leading initiatives like those of the Cochrane Collaboration and the Joanna Briggs Institute, which require mandatory, publicly available protocols [1, 2].

It is also worth to emphasize that a prospective registry clarifies the review methods, aiming to reduce bias and increase confidence in the reported results. Although our study did not aim to assess the overall methodological quality of the included systematic reviews, previous meta‐research has demonstrated a positive association between protocol registration and review quality [14, 15]. Authors of registered reviews may have stronger methodological skills, possibly due to greater familiarity with PRISMA and PROSPERO requirements [14]. Also, registered systematic reviews were more likely to meet methodological quality standards, as assessed using the AMSTAR‐2 tool [15].

To our knowledge, this is the first meta‐research study to systematically map systematic reviews on PBM therapy, with a focus on the adequacy of protocol registration. A key strength of the study is its comprehensive assessment of a large and heterogeneous sample of systematic reviews addressing a broad range of health conditions. However, the search strategy was limited to two databases and did not include a hand search, and it may have resulted in the omission of relevant reviews not indexed or captured by the selected sources, reducing the overall comprehensiveness of the mapping. Additionally, the analysis of redundancy among systematic review topics was descriptive rather than quantitative, which may have underestimated the true extent of overlap between reviews.

Given the essential role of protocol registration in promoting transparency, reducing bias, and enhancing the overall reliability of systematic reviews, future efforts in PBM research should prioritize the widespread adoption and consistent updating of registered protocols. Journals, funding agencies, and academic institutions should also reinforce the requirement of review protocol registration and consider implementing policies that recommend protocol submission as part of the review or publication process. Strengthening these practices has direct implications for clinicians, researchers, and policymakers because, without prospectively registered and regularly updated protocols, the reliability and reproducibility of PBM evidence may be compromised, increasing the risk of biased or redundant conclusions. Promoting prospective registration, transparent reporting of deviations, and avoidance of overlapping review topics will enhance the credibility of PBM research and support the development of more trustworthy clinical guidelines and policy decisions regarding therapeutic PBM applications.

## 5 Conclusion

This meta‐research study found that fewer than half of PBM systematic reviews had publicly registered protocols, revealing persistent gaps in transparency and methodological rigor. Strengthening prospective registration and regular protocol updates is essential to improve the credibility and reproducibility of PBM evidence. Future research should explore how adherence to protocol registration and reporting standards influences methodological quality, redundancy, and reliability of PBM systematic reviews. Journals, funders, and research institutions can play a decisive role by requiring prospective registration, verifying protocol adherence during peer review, and promoting training in evidence synthesis methodology. These coordinated actions will advance the transparency, efficiency, and clinical relevance of future PBM research.

## Author Contributions

**Giovanna Marcilio Santos:** conceptualization, methodology, data curation and screening, writing – original draft preparation. **Giullia Carvalho Mangas Lopes:** methodology, data curation and screening, writing – original draft preparation. **Kamilla Mayr Martins Sád:** data curation and screening, writing – original draft preparation. **Giulia Almirón da Rocha Soares:** data curation and screening. **Marcela Letícia Leal Gonçalves:** formal analysis. **Sandra Kalil Bussadori:** writing – review and editing, supervision. **Elaine Marcílio Santos:** conceptualization, writing – review and editing, supervision. **Ana Luiza Cabrera Martimbianco:** conceptualization, methodology, formal analysis, writing – review and editing, supervision. All authors read and approved the final version of the manuscript.

## Conflicts of Interest

The authors declare no conflicts of interest.

## Supporting information

Online Resource 1.

Online Resource 2.

Online Resource 3.

Online Resource 4.

## Data Availability

Data sharing not applicable to this article as no datasets were generated or analysed during the current study.
