# Supplementary material for: Adherence to Protocol Registration Among Systematic Reviews on Photobiomodulation: A Meta‐Research Study
Source: J Eval Clin Pract. 2026 Jan 8;32(1):e70346. doi: 10.1111/jep.70346 (PMC12783942; doi:10.1111/jep.70346)
Supplement: Supplementary file 1 — Online Resource 1. [file JEP-32-0-s001.docx]

Supplementary file 1. Search strategies.

| **Database** | **Search strategy** | **Results** |
| --- | --- | --- |
| MEDLINE (via PubMed) | #1 "Low-Level Light Therapy" [Mesh] OR (Low-Level Light Therapy) OR (Light Therapies, Low-Level) OR (Light Therapy, Low-Level) OR (Low Level Light Therapy) OR (Low-Level Light Therapies) OR (Therapies, Low-Level Light) OR (Therapy, Low-Level Light) OR (Photobiomodulation Therapy) OR (Photobiomodulation Therapies) OR (Therapies, Photobiomodulation) OR (Therapy, Photobiomodulation) OR (LLLT) OR (Laser Therapy, Low-Level) OR (Laser Therapies, Low-Level) OR (Laser Therapy, Low Level) OR (Low-Level Laser Therapies) OR (Laser Irradiation, Low-Power) OR (Irradiation, Low-Power Laser) OR (Laser Irradiation, Low Power) OR (Low-Power Laser Therapy) OR (Low Power Laser Therapy) OR (Laser Therapy, Low-Power) OR (Laser Therapies, Low-Power) OR (Laser Therapy, Low Power) OR (Low-Power Laser Therapies) OR (Low-Level Laser Therapy) OR (Low Level Laser Therapy) OR (Low-Power Laser Irradiation) OR (Low Power Laser Irradiation) OR (Laser Biostimulation) OR (Laser Phototherapy) OR (Phototherapy, Laser)  #2 systematic[sb]  #3 #1 AND #2 | 920 |
| Epistemonikos | Photobiomodulation OR "Low-Level Light Therapy" | 328 |
| Total | | 1,248 |
