# Supplementary material for: Adherence to Protocol Registration Among Systematic Reviews on Photobiomodulation: A Meta‐Research Study
Source: J Eval Clin Pract. 2026 Jan 8;32(1):e70346. doi: 10.1111/jep.70346 (PMC12783942; doi:10.1111/jep.70346)
Supplement: Supplementary file 2 — Online Resource 2. [file JEP-32-0-s004.docx]

Supplementary material 2. Excluded studies after full-text analysis

| Andrade Filho 2024 | Included *in vitro* studies |
| --- | --- |
| Bathini 2022 | Included *in vivo/vitro* studies |
| da Silva | Included *in vitro* studies |
| Rosso 2018 | Included *in vivo* studies |
| Wan 2020 | Included *in vitro* studies |
| Yi 2017 | Included *in vivo* studies |

**References**

Andrade Filho VO, Amarante MOC, Gonzalez-Lima F, Gomes da Silva S, Cardoso FDS. Systematic review of photobiomodulation for multiple sclerosis. Front Neurol. 2024 Sep 12;15:1465621. doi: 10.3389/fneur.2024.1465621.

Bathini M, Raghushaker CR, Mahato KK. The Molecular Mechanisms of Action of Photobiomodulation Against Neurodegenerative Diseases: A Systematic Review. Cell Mol Neurobiol. 2022 May;42(4):955-971. doi: 10.1007/s10571-020-01016-9.

da Silva JL, Silva-de-Oliveira AFS, Andraus RAC, Maia LP. Effects of low level laser therapy in cancer cells-a systematic review of the literature. Lasers Med Sci. 2020 Apr;35(3):523-529. doi: 10.1007/s10103-019-02824-2.

Rosso MPO, Buchaim DV, Kawano N, Furlanette G, Pomini KT, Buchaim RL. Photobiomodulation Therapy (PBMT) in Peripheral Nerve Regeneration: A Systematic Review. Bioengineering (Basel). 2018;5(2):44. Published 2018 Jun 9. doi:10.3390/bioengineering5020044

Wan Z, Zhang P, Lv L, Zhou Y. NIR light-assisted phototherapies for bone-related diseases and bone tissue regeneration: A systematic review. Theranostics. 2020;10(25):11837-11861. Published 2020 Sep 26. doi:10.7150/thno.49784

Yi J, Xiao J, Li H, Li Y, Li X, Zhao Z. Effectiveness of adjunctive interventions for accelerating orthodontic tooth movement: a systematic review of systematic reviews. J Oral Rehabil. 2017;44(8):636-654. doi:10.1111/joor.12509
