# Supplementary material for: Adherence to Protocol Registration Among Systematic Reviews on Photobiomodulation: A Meta‐Research Study
Source: J Eval Clin Pract. 2026 Jan 8;32(1):e70346. doi: 10.1111/jep.70346 (PMC12783942; doi:10.1111/jep.70346)
Supplement: Supplementary file 3 — Online Resource 3. [file JEP-32-0-s003.docx]

Supplementary material 3. Included systematic review references.

1. Afifi L, Maranda EL, Zarei M, Delcanto GM, Falto-Aizpurua L, Kluijfhout WP, Jimenez JJ. Low-level laser therapy as a treatment for androgenetic alopecia. Lasers Surg Med. 2017 Jan;49(1):27-39. doi: 10.1002/lsm.22512.
2. Aguiar BRL, Guerra ENS, Normando AGC, Martins CC, Reis PEDD, Ferreira EB. Effectiveness of photobiomodulation therapy in radiation dermatitis: A systematic review and meta-analysis. Crit Rev Oncol Hematol. 2021 Jun;162:103349. doi: 10.1016/j.critrevonc.2021.103349.
3. Aguirra P, do Nascimento AP, Casonatto J, Ribeiro AS, Pacagnelli FL, de Oliveira RG, Aguiar AF. A systematic review and meta-analysis of the acute effects of photobiomodulation therapy on the maximum number of repetitions in resistance exercise in young adults. Lasers Med Sci. 2025 Apr 10;40(1):184. doi: 10.1007/s10103-025-04441-8. PMID: 40205065.
4. Ahmad SA, Hasan S, Saeed S, Khan A, Khan M. Low-level laser therapy in temporomandibular joint disorders: a systematic review. J Med Life. 2021 Mar-Apr;14(2):148-164. doi: 10.25122/jml-2020-0169.
5. Ahmad MA, A Hamid MS, Yusof A. Effects of low-level and high-intensity laser therapy as adjunctive to rehabilitation exercise on pain, stiffness and function in knee osteoarthritis: a systematic review and meta-analysis. Physiotherapy. 2022 Mar;114:85-95. doi: 10.1016/j.physio.2021.03.011.
6. Ahmed MK, Jafer M, Nayeem M, Hussain Moafa I, Quadri MFA, Gopalaiah H, et al. Low-Level Laser Therapy and Topical Medications for Treating Aphthous Ulcers: A Systematic Review. J Multidiscip Healthc. 2020 Nov 18;13:1595-1605. doi: 10.2147/JMDH.S281495. PMID: 33239881; PMCID: PMC7680689.
7. Akram Z, Abduljabbar T, Sauro S, Daood U. Effect of photodynamic therapy and laser alone as adjunct to scaling and root planing on gingival crevicular fluid inflammatory proteins in periodontal disease: A systematic review. Photodiagnosis Photodyn Ther. 2016 Dec;16:142-153. doi: 10.1016/j.pdpdt.2016.09.004.
8. Akram Z, Abduljabbar T, Vohra F, Javed F. Efficacy of low-level laser therapy compared to steroid therapy in the treatment of oral lichen planus: A systematic review. J Oral Pathol Med. 2018 Jan;47(1):11-17. doi: 10.1111/jop.12619.
9. Akram Z, Vohra F, Javed F. Low-level laser therapy as an adjunct to connective tissue graft procedure in the treatment of gingival recession defects: A systematic review and meta-analysis. J Esthet Restor Dent. 2018 Jul;30(4):299-306. doi: 10.1111/jerd.12377.
10. Al-Maweri SA, Javed F, Kalakonda B, AlAizari NA, Al-Soneidar W, Al-Akwa A. Efficacy of low level laser therapy in the treatment of burning mouth syndrome: A systematic review. Photodiagnosis Photodyn Ther. 2017 Mar;17:188-193. doi: 10.1016/j.pdpdt.2016.11.017.
11. Al-Maweri SA, Kalakonda B, Al-Soneidar WA, Al-Shamiri HM, Alakhali MS, Alaizari N. Efficacy of low-level laser therapy in management of symptomatic oral lichen planus: a systematic review. Lasers Med Sci. 2017 Aug;32(6):1429-1437. doi: 10.1007/s10103-017-2233-7.
12. Al-Maweri SA, Kalakonda B, AlAizari NA, Al-Soneidar WA, Ashraf S, Abdulrab S, Al-Mawri ES. Efficacy of low-level laser therapy in management of recurrent herpes labialis: a systematic review. Lasers Med Sci. 2018 Sep;33(7):1423-1430. doi: 10.1007/s10103-018-2542-5.
13. AlShahrani I, Togoo RA, Hosmani J, Alhaizaey A. Photobiomodulation in acceleration of orthodontic tooth movement: A systematic review and meta analysis. Complement Ther Med. 2019 Dec;47:102220. doi: 10.1016/j.ctim.2019.102220.
14. Al-Shibani N. Low-intensity laser for harvesting palatal graft for the treatment of gingival recession: A systematic review. J Investig Clin Dent. 2019 Feb;10(1):e12368. doi: 10.1111/jicd.12368.
15. Al-Zainal Z, Farid Ashraf S, Gopinath D. Clinical efficacy of lasers in the management of recurrent aphthous ulcers of oral cavity: a systematic review of randomized control trials. Lasers Med Sci. 2025;40(1):49. Published 2025 Jan 28. doi:10.1007/s10103-024-04268-9.
16. Alaql YB, Alkhalaf SA, Almuqaythil BA, et al. Effectiveness of Low-Level Red Light in Controlling Myopia Progression in Children: A Systematic Review and Meta-Analysis. Cureus. 2025;17(3):e80336. Published 2025 Mar 10. doi:10.7759/cureus.80336
17. Alayat MS, Takaroni A, Elsodany AM, AlMatrafi NA, Subahi MS, Battecha KH. Effectiveness of photobiomodulation therapy in the treatment of patients with an ankle sprain: a systematic review and meta-analysis. Lasers Med Sci. 2024;39(1):116. Published 2024 Apr 26. doi:10.1007/s10103-024-04063-6
18. Albaker AM, ArRejaie AS, Alrabiah M, Abduljabbar T. Effect of photodynamic and laser therapy in the treatment of peri-implant mucositis: A systematic review. Photodiagnosis Photodyn Ther. 2018 Mar;21:147-152. doi: 10.1016/j.pdpdt.2017.11.011.
19. Allameh F, Javadi A, Dadkhahfar S, Naeeji Z, Moridi A, Tadayon N, Alahyari S. A Systematic Review of Elective Laser Therapy during Pregnancy. J Lasers Med Sci. 2021 Sep 19;12:e50. doi: 10.34172/jlms.2021.50. PMID: 34733773; PMCID: PMC8558736.
20. Alonaizan FA, AlFawaz YF. Is phototherapy effective in the management of post-operative endodontic pain? A systematic review of randomized controlled clinical trials. Photodiagnosis Photodyn Ther. 2019 Jun;26:53-58. doi: 10.1016/j.pdpdt.2019.03.003. Epub 2019 Mar 2. PMID: 30836213.
21. Altuhafy M, Baig V, Jabr L, Khan J. The efficacy of photobiomodulation on dental injection pain: a systematic review of randomized clinical trials. J Dent Anesth Pain Med. 2024;24(3):145-159. doi:10.17245/jdapm.2024.24.3.145
22. Álvarez-Martínez M, Borden G. A systematic review on whole-body photobiomodulation for exercise performance and recovery. Lasers Med Sci. 2025;40(1):55. Published 2025 Jan 30. doi:10.1007/s10103-025-04318-w
23. Amiri P, Fekrazad R. Efficacy of photobiomodulation therapy on Bell's palsy symptoms: a systematic review. Lasers Med Sci. 2024;39(1):288. Published 2024 Dec 4. doi:10.1007/s10103-024-04240-7
24. Anagnostaki E, Mylona V, Parker S, Lynch E, Grootveld M. Systematic Review on the Role of Lasers in Endodontic Therapy: Valuable Adjunct Treatment? Dent J (Basel). 2020 Jul 1;8(3):63. doi: 10.3390/dj8030063. PMID: 32630217; PMCID: PMC7559699.
25. Anju M, Ummer VS, Maiya AG, Hande M. Low level laser therapy for the patients with painful diabetic peripheral neuropathy - A systematic review. Diabetes Metab Syndr. 2019 Jul-Aug;13(4):2667-2670. doi: 10.1016/j.dsx.2019.07.035. Epub 2019 Jul 13. PMID: 31405692.
26. Anschau F, Webster J, Capra MEZ, de Azeredo da Silva ALF, Stein AT. Efficacy of low-level laser for treatment of cancer oral mucositis: a systematic review and meta-analysis. Lasers Med Sci. 2019 Aug;34(6):1053-1062. doi: 10.1007/s10103-019-02722-7. Epub 2019 Feb 7. PMID: 30729351.
27. Austin E, Mamalis A, Ho D, Jagdeo J. Laser and light-based therapy for cutaneous and soft-tissue metastases of malignant melanoma: a systematic review. Arch Dermatol Res. 2017 May;309(4):229-242. doi: 10.1007/s00403-017-1720-9. Epub 2017 Mar 17. PMID: 28314913.
28. Ayen-Rodriguez A, Naranjo-Diaz MJ, Ruiz-Villaverde R. Laser Therapy for the Treatment of Actinic Cheilitis: A Systematic Review. Int J Environ Res Public Health. 2022 Apr 11;19(8):4593. doi: 10.3390/ijerph19084593. PMID: 35457467; PMCID: PMC9028420.
29. Bai Y, Liu L, Yuan H, Li J, Tang Y, Pu C, Han P. Safety and efficacy of transurethral laser therapy for bladder cancer: a systematic review and meta-analysis. World J Surg Oncol. 2014 Sep 25;12:301. doi: 10.1186/1477-7819-12-301. PMID: 25256383; PMCID: PMC4190332.
30. Bakdach WMM, Hadad R. Effectiveness of low-level laser therapy in accelerating the orthodontic tooth movement: A systematic review and meta-analysis. Dent Med Probl. 2020 Jan-Mar;57(1):73-94. doi: 10.17219/dmp/112446. PMID: 32314880.
31. Baxter GD, Liu L, Petrich S, Gisselman AS, Chapple C, Anders JJ, Tumilty S. Low level laser therapy (Photobiomodulation therapy) for breast cancer-related lymphedema: a systematic review. BMC Cancer. 2017 Dec 7;17(1):833. doi: 10.1186/s12885-017-3852-x. PMID: 29216916; PMCID: PMC5719569.
32. Bekhet AH, Ragab B, Abushouk AI, Elgebaly A, Ali OI. Efficacy of low-level laser therapy in carpal tunnel syndrome management: a systematic review and meta-analysis. Lasers Med Sci. 2017 Aug;32(6):1439-1448. doi: 10.1007/s10103-017-2234-6. Epub 2017 Jun 5. PMID: 28580494.
33. Bittencourt MA, Paranhos LR, Martins-Filho PR. Low-level laser therapy for treatment of neurosensory disorders after orthognathic surgery: A systematic review of randomized clinical trials. Med Oral Patol Oral Cir Bucal. 2017 Nov 1;22(6):780-787. doi: 10.4317/medoral.21968. PMID: 29053658; PMCID: PMC5813998.
34. Bjordal JM, Couppé C, Chow RT, Tunér J, Ljunggren EA. A systematic review of low level laser therapy with location-specific doses for pain from chronic joint disorders. Aust J Physiother. 2003;49(2):107-16. doi: 10.1016/s0004-9514(14)60127-6. PMID: 12775206.
35. Bjordal JM, Johnson MI, Lopes-Martins RA, Bogen B, Chow R, Ljunggren AE. Short-term efficacy of physical interventions in osteoarthritic knee pain. A systematic review and meta-analysis of randomised placebo-controlled trials. BMC Musculoskelet Disord. 2007 Jun 22;8:51. doi: 10.1186/1471-2474-8-51. PMID: 17587446; PMCID: PMC1931596.
36. Bjordal JM, Lopes-Martins RA, Joensen J, Couppe C, Ljunggren AE, Stergioulas A, Johnson MI. A systematic review with procedural assessments and meta-analysis of low level laser therapy in lateral elbow tendinopathy (tennis elbow). BMC Musculoskelet Disord. 2008 May 29;9:75. doi: 10.1186/1471-2474-9-75. PMID: 18510742; PMCID: PMC2442599.
37. Bjordal JM, Bensadoun RJ, Tunèr J, Frigo L, Gjerde K, Lopes-Martins RA. A systematic review with meta-analysis of the effect of low-level laser therapy (LLLT) in cancer therapy-induced oral mucositis. Support Care Cancer. 2011 Aug;19(8):1069-77. doi: 10.1007/s00520-011-1202-0. Epub 2011 Jun 10. PMID: 21660670.
38. Borsa PA, Larkin KA, True JM. Does phototherapy enhance skeletal muscle contractile function and postexercise recovery? A systematic review. J Athl Train. 2013 Jan-Feb;48(1):57-67. doi: 10.4085/1062-6050-48.1.12. PMID: 23672326; PMCID: PMC3554033.
39. Brignardello-Petersen R, Carrasco-Labra A, Araya I, Yanine N, Beyene J, Shah PS. Is adjuvant laser therapy effective for preventing pain, swelling, and trismus after surgical removal of impacted mandibular third molars? A systematic review and meta-analysis. J Oral Maxillofac Surg. 2012 Aug;70(8):1789-801. doi: 10.1016/j.joms.2012.01.008. Epub 2012 Mar 6. PMID: 22398186.
40. Burger M, Kriel R, Damon A, Abel A, Bansda A, Wakens M, Ernstzen D. The effectiveness of low-level laser therapy on pain, self-reported hand function, and grip strength compared to placebo or "sham" treatment for adults with carpal tunnel syndrome: A systematic review. Physiother Theory Pract. 2017 Mar;33(3):184-197. doi: 10.1080/09593985.2017.1282999. Epub 2017 Mar 8. PMID: 28272964.
41. Calarga CC, Cotomácio CC, Simões A. Photobiomodulation for oral mucositis management in pediatric patients: a systematic review. Lasers Med Sci. 2024;39(1):272. Published 2024 Nov 10. doi:10.1007/s10103-024-04221-w
42. Camolesi GCV, Marichalar-Mendía X, Padín-Iruegas ME, Spanemberg JC, López-López J, Blanco-Carrión A, Gándara-Vila P, Gallas-Torreira M, Pérez-Sayáns M. Efficacy of photobiomodulation in reducing pain and improving the quality of life in patients with idiopathic burning mouth syndrome. A systematic review and meta-analysis. Lasers Med Sci. 2022 Jun;37(4):2123-2133. doi: 10.1007/s10103-022-03518-y. Epub 2022 Feb 5. PMID: 35122543; PMCID: PMC9148274.
43. Camolesi GCV, Silva FFVE, Aulestia-Viera PV, Marichalar-Mendía X, Gándara-Vila P, Pérez-Sayáns M. Is The Photobiomodulation Therapy Effective In Controlling Post-Surgical Side Effects After The Extraction Of Mandibular Third Molars? A Systematic Review And Meta-Analysis. J Evid Based Dent Pract. 2024;24(2):101983. doi:10.1016/j.jebdp.2024.101983
44. Campos TM, do Prado Tavares Silva CA, Sobral APT, Sobral SS, Rodrigues MFSD, Bussadori SK, Fernandes KPS, Mesquita-Ferrari RA, Horliana ACRT, Motta LJ. Photobiomodulation in oral mucositis in patients with head and neck cancer: a systematic review and meta-analysis followed by a cost-effectiveness analysis. Support Care Cancer. 2020 Dec;28(12):5649-5659. doi: 10.1007/s00520-020-05613-8. Epub 2020 Jul 14. PMID: 32666214.
45. Carneiro AMP, Barros APO, de Oliveira RP, de Paula BLF, Silva AM, de Melo Alencar C, Silva CM. The effect of photobiomodulation using low-level laser therapy on tooth sensitivity after dental bleaching: a systematic review. Lasers Med Sci. 2022 Sep;37(7):2791-2804. doi: 10.1007/s10103-022-03578-0. Epub 2022 May 21. PMID: 35597839.
46. Cerqueira NM, Altube LG, Gambarini L, Borsatto MC, Corona SAM. Laser therapy in Molar-Incisor Hypomineralization (MIH) teeth treatment of hypersensitivity: a systematic review. Lasers Med Sci. 2025;40(1):11. Published 2025 Jan 7. doi:10.1007/s10103-024-04280-z.
47. Chang WD, Wu JH, Yang WJ, Jiang JA. Therapeutic effects of low-level laser on lateral epicondylitis from differential interventions of Chinese-Western medicine: systematic review. Photomed Laser Surg. 2010 Jun;28(3):327-36. doi: 10.1089/pho.2009.2558. PMID: 19874256.
48. Chaple Gil A, Díaz L, Von Marttens A, et al. The efficacy of low-level laser therapy in oral surgery: A systematic review of randomized controlled trials. Photodiagnosis Photodyn Ther. 2025;53:104594. doi:10.1016/j.pdpdt.2025.104594
49. Chen HY, Tsai HH, Tam KW, Huang TW. Effects of photobiomodualtion therapy on breast cancer-related lymphoedema: A systematic review and meta-analysis of randomised controlled trials. Complement Ther Med. 2019 Dec;47:102200. doi: 10.1016/j.ctim.2019.102200. Epub 2019 Sep 24. PMID: 31780036.
50. Chen Y, Liu C, Chen X, Mo A. Clinical evidence of photobiomodulation therapy (PBMT) on implant stability and success: a systematic review and meta-analysis. BMC Oral Health. 2019 May 7;19(1):77. doi: 10.1186/s12903-019-0779-4. PMID: 31064350; PMCID: PMC6505209.
51. Chen Y, Chen XL, Zou XL, Chen SZ, Zou J, Wang Y. Efficacy of low-level laser therapy in pain management after root canal treatment or retreatment: a systematic review. Lasers Med Sci. 2019 Sep;34(7):1305-1316. doi: 10.1007/s10103-019-02793-6. Epub 2019 May 1. PMID: 31044364.
52. Chen CH, Huang CY, Chang CY, Cheng YF. Efficacy of Low-Level Laser Therapy for Tinnitus: A Systematic Review with Meta-Analysis and Trial Sequential Analysis. Brain Sci. 2020 Dec 2;10(12):931. doi: 10.3390/brainsci10120931. PMID: 33276501; PMCID: PMC7761002.
53. Chen J, Chen A, Zhang J, Wang F, Fang Q, He Z, Chen X, Ma W, Hu F. Efficacy and safety of laser combination therapy and laser alone therapy for keloid: a systematic review and meta-analysis. Lasers Med Sci. 2022 Mar;37(2):1127-1138. doi: 10.1007/s10103-021-03364-4. Epub 2021 Jul 20. PMID: 34283306.
54. Chen Y, Xiong R, Yang S, et al. Safety of repeated low-level red-light therapy for myopia: A systematic review. Asia Pac J Ophthalmol (Phila). 2024;13(6):100124. doi:10.1016/j.apjo.2024.100124.
55. Chen B, Lin Z, Zou S, Huang C, Liu Y, Xu S. Intervention effects of low-level laser therapy (LLLT) on grade I-II ulcers in diabetic foot patients: A meta-analysis. Wound Repair Regen. 2025;33(2):e70021. doi:10.1111/wrr.70021
56. Chen, KY, Lee, HK, Chan, HC, et al. Is Multiwavelength Photobiomodulation Effective and Safe for Age-Related Macular Degeneration? A Systematic Review and Meta-Analysis. Ophthalmol Ther 14, 969–987 (2025). [https://doi.org/10.1007/s40123-025-01119-](https://doi.org/10.1007/s40123-025-01119-w)w.
57. Chow RT, Barnsley L. Systematic review of the literature of low-level laser therapy (LLLT) in the management of neck pain. Lasers Surg Med. 2005 Jul;37(1):46-52. doi: 10.1002/lsm.20193. PMID: 15954117.
58. Clijsen R, Brunner A, Barbero M, Clarys P, Taeymans J. Effects of low-level laser therapy on pain in patients with musculoskeletal disorders: a systematic review and meta-analysis. Eur J Phys Rehabil Med. 2017 Aug;53(4):603-610. doi: 10.23736/S1973-9087.17.04432-X. Epub 2017 Jan 30. PMID: 28145397.
59. Coluzzi D, Anagnostaki E, Mylona V, Parker S, Lynch E. Do Lasers Have an Adjunctive Role in Initial Non-Surgical Periodontal Therapy? A Systematic Review. Dent J (Basel). 2020 Aug 16;8(3):93. doi: 10.3390/dj8030093. PMID: 32824321; PMCID: PMC7558016.
60. Costa ACF, Maia TAC, de Barros Silva PG, Abreu LG, Gondim DV, Santos PCF. Effects of low-level laser therapy on the orthodontic mini-implants stability: a systematic review and meta-analysis. Prog Orthod. 2021 Feb 15;22(1):6. doi: 10.1186/s40510-021-00350-y. PMID: 33586080; PMCID: PMC7882650.
61. Cronshaw M, Parker S, Anagnostaki E, Lynch E. Systematic Review of Orthodontic Treatment Management with Photobiomodulation Therapy. Photobiomodul Photomed Laser Surg. 2019 Dec;37(12):862-868. doi: 10.1089/photob.2019.4702. Epub 2019 Nov 22. PMID: 31755850.
62. Cronshaw M, Parker S, Anagnostaki E, Mylona V, Lynch E, Grootveld M. Photobiomodulation Dose Parameters in Dentistry: A Systematic Review and Meta-Analysis. Dent J (Basel). 2020 Oct 6;8(4):114. doi: 10.3390/dj8040114. PMID: 33036145; PMCID: PMC7711492.
63. Davoudi A, Amrolahi M, Khaki H. Effects of laser therapy on patients who underwent rapid maxillary expansion; a systematic review. Lasers Med Sci. 2018 Aug;33(6):1387-1395. doi: 10.1007/s10103-018-2545-2. Epub 2018 Jun 12. PMID: 29948453.
64. Davoudi A, Ebadian B, Nosouhian S. Role of laser or photodynamic therapy in treatment of denture stomatitis: A systematic review. J Prosthet Dent. 2018 Oct;120(4):498-505. doi: 10.1016/j.prosdent.2018.01.003. Epub 2018 May 25. PMID: 29807743.
65. Dawdy J, Halladay J, Carrasco-Labra A, Araya I, Yanine N, Brignardello-Petersen R. Efficacy of adjuvant laser therapy in reducing postsurgical complications after the removal of impacted mandibular third molars: A systematic review update and meta-analysis. J Am Dent Assoc. 2017 Dec;148(12):887-902.e4. doi: 10.1016/j.adaj.2017.06.043. Epub 2017 Oct 5. PMID: 28987483.
66. da Silva Mira PC, Biagini ACSCF, Gomes MG, Galo R, Corona SAM, Borsatto MC. Laser acupuncture to reduce temporomandibular disorder (TMD) symptoms: systematic review and meta-analysis. Lasers Med Sci. 2024;39(1):66. Published 2024 Feb 20. doi:10.1007/s10103-024-03999-z
67. de Arruda JAA, Sampaio GC, de Sena ACVP, Schuch LF, Ribeiro JP, Martins MD, Silva TA, Mesquita RA, Abreu LG. Does photobiomodulation therapy improve the postoperative outcomes of tonsillectomy? A systematic review and meta-analysis. J Lasers Med Sci. 2022 Feb 16;13:e7. doi: 10.34172/jlms.2022.07. PMID: 35642234; PMCID: PMC9131299.
68. de Barros DD, Dos Santos Barros Catão JS, Ferreira ACD, Simões TMS, Almeida RAC, de Vasconcelos Catão MHC. Low-level laser therapy is effective in controlling postoperative pain in lower third molar extractions: a systematic review and meta-analysis. Lasers Med Sci. 2022 Jul;37(5):2363-2377. doi: 10.1007/s10103-021-03470-3. Epub 2022 Jan 11. Erratum in: Lasers Med Sci. 2022 Jun 11;: PMID: 35013845.
69. de Carvalho MM, Hidalgo MAR, Scarel-Caminaga RM, Ribeiro Junior NV, Sperandio FF, Pigossi SC, de Carli ML. Photobiomodulation of gingival lesions resulting from autoimmune diseases: systematic review and meta-analysis. Clin Oral Investig. 2022 May;26(5):3949-3964. doi: 10.1007/s00784-021-04362-0. Epub 2022 Jan 13. PMID: 35024960; PMCID: PMC8755514.
70. de Lima VHS, de Oliveira-Neto OB, da Hora Sales PH, da Silva Torres T, de Lima FJC. Effectiveness of low-level laser therapy for oral mucositis prevention in patients undergoing chemoradiotherapy for the treatment of head and neck cancer: A systematic review and meta-analysis. Oral Oncol. 2020 Mar;102:104524. doi: 10.1016/j.oraloncology.2019.104524. Epub 2020 Feb 13. PMID: 32062592.
71. de Marchi T, Ferlito JV, Ferlito MV, Salvador M, Leal-Junior ECP. Can Photobiomodulation Therapy (PBMT) Minimize Exercise-Induced Oxidative Stress? A Systematic Review and Meta-Analysis. Antioxidants (Basel). 2022 Aug 27;11(9):1671. doi: 10.3390/antiox11091671. PMID: 36139746; PMCID: PMC9495825.
72. de Oliveira FJD, Brasil GMLC, Araújo Soares GP, Fernandes Paiva DF, de Assis de Souza Júnior F. Use of low-level laser therapy to reduce postoperative pain, edema, and trismus following third molar surgery: A systematic review and meta-analysis. J Craniomaxillofac Surg. 2021 Nov;49(11):1088-1096. doi: 10.1016/j.jcms.2021.06.006. Epub 2021 Jun 22. PMID: 34217567.
73. de Pedro M, López-Pintor RM, de la Hoz-Aizpurua JL, Casañas E, Hernández G. Efficacy of Low-Level Laser Therapy for the Therapeutic Management of Neuropathic Orofacial Pain: A Systematic Review. J Oral Facial Pain Headache. 2020 Winter;34(1):13–30. doi: 10.11607/ofph.2310. Epub 2019 Jul 24. PMID: 31339967.
74. Deana NF, Zaror C, Sandoval P, Alves N. Effectiveness of Low-Level Laser Therapy in Reducing Orthodontic Pain: A Systematic Review and Meta-Analysis. Pain Res Manag. 2017;2017:8560652. doi: 10.1155/2017/8560652. Epub 2017 Sep 27. PMID: 29089818; PMCID: PMC5635293.
75. Delaney SW, Zhang P. Systematic review of low-level laser therapy for adult androgenic alopecia. J Cosmet Laser Ther. 2018 Aug;20(4):229-236. doi: 10.1080/14764172.2017.1400170. Epub 2017 Dec 29. PMID: 29286826.
76. Díaz L, Restelli L, Valencia E, et al. Effectiveness of low-level laser therapy on temporomandibular disorders. A systematic review of randomized clinical trials. Photodiagnosis Photodyn Ther. 2025;53:104558. doi:10.1016/j.pdpdt.2025.104558.
77. Díaz L, Basualdo J, Chaple-Gil A, et al. Effectiveness of low-level laser therapy in patients with maxillofacial neuropathies. A systematic review of randomized controlled trials. Photodiagnosis Photodyn Ther. 2025;52:104516. doi:10.1016/j.pdpdt.2025.104516.
78. Dos Santos KW, Rech RS, Wendland EMDR, Hilgert JB. Rehabilitation strategies in maxillofacial trauma: systematic review and meta-analysis. Oral Maxillofac Surg. 2020 Mar;24(1):1-10. doi: 10.1007/s10006-019-00808-8. Epub 2019 Dec 4. PMID: 31802332.
79. Dos Santos SA, Sampaio LM, Caires JR, Fernandes GHC, Marsico A, Serra AJ, Leal-Junior EC, de Carvalho PTC. Parameters and Effects of Photobiomodulation in Plantar Fasciitis: A Meta-Analysis and Systematic Review. Photobiomodul Photomed Laser Surg. 2019 Jun;37(6):327-335. doi: 10.1089/photob.2018.4588. Epub 2019 May 20. PMID: 31107161.
80. Ebrahimi P, Hadilou M, Naserneysari F, Dolatabadi A, Tarzemany R, Vahed N, Nikniaz L, Fekrazad R, Gholami L. Effect of photobiomodulation in secondary intention gingival wound healing-a systematic review and meta-analysis. BMC Oral Health. 2021 May 13;21(1):258. doi: 10.1186/s12903-021-01611-2. PMID: 33985492; PMCID: PMC8120828.
81. Elmsmari F, Shujaie H, Alzaabi R, et al. Lasers efficacy in pain management after primary and secondary endodontic treatment: a systematic review and meta-analysis of randomized clinical trials. Sci Rep. 2024;14(1):26028. Published 2024 Oct 29. doi:10.1038/s41598-024-74998-x
82. Escudero JSB, Perez MGB, de Oliveira Rosso MP, Buchaim DV, Pomini KT, Campos LMG, Audi M, Buchaim RL. Photobiomodulation therapy (PBMT) in bone repair: A systematic review. Injury. 2019 Nov;50(11):1853-1867. doi: 10.1016/j.injury.2019.09.031. Epub 2019 Sep 21. PMID: 31585673.
83. Ezzati K, Laakso EL, Salari A, Hasannejad A, Fekrazad R, Aris A. The Beneficial Effects of High-Intensity Laser Therapy and Co-Interventions on Musculoskeletal Pain Management: A Systematic Review. J Lasers Med Sci. 2020 Winter;11(1):81-90. doi: 10.15171/jlms.2020.14. Epub 2020 Jan 18. PMID: 32099632; PMCID: PMC7008744.
84. Farzan A, Khaleghi K. The Effectiveness of Low-Level Laser Therapy in Pain Induced by Orthodontic Separator Placement: A Systematic Review. J Lasers Med Sci. 2021 Jun 24;12:e29. doi: 10.34172/jlms.2021.29. PMID: 34733752; PMCID: PMC8558704.
85. Farzan A, Khaleghi K, Pirayesh Z. Effect of Low-Level Laser Therapy on Bone Formation in Rapid Palatal Expansion: A Systematic Review. J Lasers Med Sci. 2022 Mar 23;13:e13. doi: 10.34172/jlms.2022.13. PMID: 35996494; PMCID: PMC9392875.
86. Ferrillo M, Ammendolia A, Paduano S, Calafiore D, Marotta N, Migliario M, Fortunato L, Giudice A, Michelotti A, de Sire A. Efficacy of rehabilitation on reducing pain in muscle-related temporomandibular disorders: A systematic review and meta-analysis of randomized controlled trials. J Back Musculoskelet Rehabil. 2022;35(5):921-936. doi: 10.3233/BMR-210236. PMID: 35213347.
87. Figueiredo AL, Lins L, Cattony AC, Falcão AF. Laser therapy in the control of oral mucositis: a meta-analysis. Rev Assoc Med Bras (1992). 2013 Sep-Oct;59(5):467-74. English, Portuguese. doi: 10.1016/j.ramb.2013.08.003. Epub 2013 Oct 10. PMID: 24119379.
88. Firoozi P, Keyhan SO, Kim SG, Fallahi HR. Effectiveness of low-level laser therapy on recovery from neurosensory disturbance after sagittal split ramus osteotomy: a systematic review and meta-analysis. Maxillofac Plast Reconstr Surg. 2020 Dec 17;42(1):41. doi: 10.1186/s40902-020-00285-0. PMID: 33331972; PMCID: PMC7746795.
89. Flemming KA, Cullum NA, Nelson EA. A systematic review of laser therapy for venous leg ulcers. J Wound Care. 1999;8(3):111-114. doi:10.12968/jowc.1999.8.3.25848
90. Fornaini C, Arany P, Rocca JP, Merigo E. Photobiomodulation in Pediatric Dentistry: A Current State-of-the-Art. Photobiomodul Photomed Laser Surg. 2019 Dec;37(12):798-813. doi: 10.1089/photob.2019.4722. PMID: 31873064.
91. Franke TPC, Koes BW, Geelen SJG, Huisstede BMA. Do Patients with Carpal Tunnel Syndrome Benefit from Low-Level Laser Therapy? A Systematic Review of Randomized Controlled Trials. Arch Phys Med Rehabil. 2017 June 16; 99(6):1650-1659. doi: 10.1016/j.apmr.2017.06.002. Available from <https://www.archives-pmr.org/article/S0003-9993(17)30391-X/fulltext>
92. Gaitero MVC, de Mira TAA, Gondim EJL, do Nascimento SL, Surita FG. Low-level laser therapy for nipple trauma and pain during breastfeeding: systematic review and meta-analysis. Rev Bras Ginecol Obstet. 2025;47:e-rbgo3. Published 2025 Mar 17. doi:10.61622/rbgo/2025rbgo3
93. Galiano-Castillo N, Liu L, Lozano-Lozano M, Tumilty S, Cantarero-Villanueva I, Baxter GD. Acute and cumulative benefits of Photobiomodulation for xerostomia: A systematic review and meta-analysis. Oral Dis. 2021;27(5):1115-1126. doi:10.1111/odi.13648
94. Garola F, Gilligan G, Panico R, Leonardi N, Piemonte E. Clinical management of alveolar osteitis. A systematic review. Med Oral Patol Oral Cir Bucal. 2021 Nov 1;26 (6):e691-702. doi:10.4317/medoral.24256.
95. Gavish L, Houreld NN. Therapeutic Efficacy of Home-Use Photobiomodulation Devices: A Systematic Literature Review. Photobiomodul Photomed Laser Surg. 2019;37(1):4-16. doi:10.1089/photob.2018.4512
96. Ge MK, He WL, Chen J, Wen C, Yin X, Hu ZA et al. Efficacy of low-level laser therapy for accelerating tooth movement during orthodontic treatment: a systematic review and meta-analysis. Lasers Med Sci. 2015 Jul;30(5):1609-18. doi: 10.1007/s10103-014-1538-z.
97. Gkantidis N, Mistakidis I, Kouskoura T, Pandis N. Effectiveness of non-conventional methods for accelerated orthodontic tooth movement: a systematic review and meta-analysis. J Dent. 2014 Oct;42(10):1300-19. doi: 10.1016/j.jdent.2014.07.013.
98. Godaert L, Dramé M. Efficacy of Photobiomodulation Therapy in Older Adults: A Systematic Review. Biomedicines. 2024;12(7):1409. Published 2024 Jun 25. doi:10.3390/biomedicines12071409
99. Golež A, Frangež I, Cankar K, Frangež HB, Ovsenik M, Nemeth L. Effects of low-level light therapy on xerostomia related to hyposalivation: a systematic review and meta-analysis of clinical trials. Lasers Med Sci. 2022 Mar;37(2):745-758. doi: 10.1007/s10103-021-03392-0.
100. Gomes AO, Martimbianco ALC, Brugnera Junior A, et al. Photobiomodulation for the Treatment of Primary Headache: Systematic Review of Randomized Clinical Trials. Life (Basel). 2022;12(1):98. Published 2022 Jan 11. doi:10.3390/life12010098
101. Gondivkar DSM, Gadbail DAR, Sarode DSC, Gondivkar DRS, Patil S, Gaikwad DRN, et al. Treatment outcomes of laser therapy in oral submucous fibrosis-a systematic review. J Oral Biol Craniofac Res. 2020 May 11;10(3):253-258. doi: 10.1016/j.jobcr.2020.05.004.
102. Gross AR, Goldsmith C, Hoving JL, Haines T, Peloso P, Aker P, et al. Cervical Overview Group. Conservative management of mechanical neck disorders: a systematic review. J Rheumatol. 2007 May;34(5):1083-102. Epub 2007 Jan 15. PMID: 17295434.
103. Gross AR, Dziengo S, Boers O, Goldsmith CH, Graham N, Lilge L, et al. Low Level Laser Therapy (LLLT) for Neck Pain: A Systematic Review and Meta-Regression. Open Orthop J. 2013 Sep 20;7:396-419. doi: 10.2174/1874325001307010396. PMID: 24155802; PMCID: PMC3802126.
104. Guimarães JS, Arcanjo FL, Leporace G, Metsavaht LF, Sena C, Moreno MVMG, et al. Effect of low-level laser therapy on pain and disability in patients with plantar fasciitis: A systematic review and meta-analysis. Musculoskelet Sci Pract. 2022 Feb;57:102478. doi: 10.1016/j.msksp.2021.102478. Epub 2021 Nov 16. PMID: 34847470.
105. Gupta AK, Bamimore MA. Factors influencing the effect of photobiomodulation in the treatment of androgenetic alopecia: A systematic review and analyses of summary-level data. Dermatol Ther. 2020 Nov;33(6):e14191. doi: 10.1111/dth.14191. Epub 2020 Sep 21. PMID: 32790116.
106. Gutiérrez-Menéndez A, Marcos-Nistal M, Méndez M, Arias JL. Photobiomodulation as a promising new tool in the management of psychological disorders: A systematic review. Neurosci Biobehav Rev. 2020 Dec;119:242-254. doi: 10.1016/j.neubiorev.2020.10.002. Epub 2020 Oct 15. PMID: 33069687.
107. Hadis MA, Zainal SA, Holder MJ, Carroll JD, Cooper PR, Milward MR, et al. The dark art of light measurement: accurate radiometry for low-level light therapy. Lasers Med Sci. 2016 May;31(4):789-809. doi: 10.1007/s10103-016-1914-y. Epub 2016 Mar 10. PMID: 26964800; PMCID: PMC4851696.
108. Haghighat S, Rezazadeh F, Sedarat H, Tabesh A, Tayebi Khorami E, Aghasadeghi K. Efficacy of Laser Therapy in Trigeminal Neuralgia: a Systematic Review. J Dent (Shiraz). 2024;25(1):17-25. Published 2024 Mar 1. doi:10.30476/dentjods.2023.95758.1889
109. Hakimiha N, Bassir SH, Romanos GE, Shamshiri AR, Moslemi N. Efficacy of photobiomodulation therapy on neurosensory recovery in patients with inferior alveolar nerve injury following oral surgical procedures: a systematic review. Quintessence Int. 2021;52(2):140-153. doi:10.3290/j.qi.a45430
110. Han M, Fang H, Li QL, Cao Y, Xia R, Zhang ZH. Effectiveness of Laser Therapy in the Management of Recurrent Aphthous Stomatitis: A Systematic Review. Scientifica (Cairo). 2016;2016:9062430. doi: 10.1155/2016/9062430. Epub 2016 Dec 18. PMID: 28078164; PMCID: PMC5203897.
111. Hanna R, Dalvi S, Bensadoun RJ, Benedicenti S. Role of Photobiomodulation Therapy in Modulating Oxidative Stress in Temporomandibular Disorders. A Systematic Review and Meta-Analysis of Human Randomised Controlled Trials. Antioxidants (Basel). 2021 Jun 25;10(7):1028. doi: 10.3390/antiox10071028. PMID: 34202292; PMCID: PMC8300797.
112. Hanna R, Dalvi S, Bensadoun RJ, Raber-Durlacher JE, Benedicenti S. Role of Photobiomodulation Therapy in Neurological Primary Burning Mouth Syndrome. A Systematic Review and Meta-Analysis of Human Randomised Controlled Clinical Trials. Pharmaceutics. 2021 Nov 2;13(11):1838. doi: 10.3390/pharmaceutics13111838. PMID: 34834253; PMCID: PMC8624276.
113. Haslerud S, Magnussen LH, Joensen J, Lopes-Martins RA, Bjordal JM. The efficacy of low-level laser therapy for shoulder tendinopathy: a systematic review and meta-analysis of randomized controlled trials. Physiother Res Int. 2015 Jun;20(2):108-25. doi: 10.1002/pri.1606. Epub 2014 Dec 2. PMID: 25450903.
114. He WL, Li CJ, Liu ZP, Sun JF, Hu ZA, Yin X, Zou SJ. Efficacy of low-level laser therapy in the management of orthodontic pain: a systematic review and meta-analysis. Lasers Med Sci. 2013 Nov;28(6):1581-9. doi: 10.1007/s10103-012-1196-y. Epub 2012 Sep 22. PMID: 23001570.
115. He WL, Yu FY, Li CJ, Pan J, Zhuang R, Duan PJ. A systematic review and meta-analysis on the efficacy of low-level laser therapy in the management of complication after mandibular third molar surgery. Lasers Med Sci. 2015 Aug;30(6):1779-88. doi: 10.1007/s10103-014-1634-0. Epub 2014 Aug 7. PMID: 25098769
116. He M, Zhang B, Shen N, Wu N, Sun J. A systematic review and meta-analysis of the effect of low-level laser therapy (LLLT) on chemotherapy-induced oral mucositis in pediatric and young patients. Eur J Pediatr. 2018 Jan;177(1):7-17. doi: 10.1007/s00431-017-3043-4. Epub 2017 Nov 11. PMID: 29128883.
117. Heiskanen V, Zadik Y, Elad S. Photobiomodulation Therapy for Cancer Treatment-Related Salivary Gland Dysfunction: A Systematic Review. Photobiomodul Photomed Laser Surg. 2020 Jun;38(6):340-347. doi: 10.1089/photob.2019.4767. Epub 2020 Feb 28. PMID: 32109187.
118. Herpich CM, Amaral AP, Leal-Junior EC, Tosato Jde P, Gomes CA, Arruda ÉE, et al. Analysis of laser therapy and assessment methods in the rehabilitation of temporomandibular disorder: a systematic review of the literature. J Phys Ther Sci. 2015 Jan;27(1):295-301. doi: 10.1589/jpts.27.295. Epub 2015 Jan 9. PMID: 25642095; PMCID: PMC4305586.
119. Hosseinpour S, Fekrazad R, Arany PR, Ye Q. Molecular impacts of photobiomodulation on bone regeneration: A systematic review. Prog Biophys Mol Biol. 2019 Dec;149:147-159. doi: 10.1016/j.pbiomolbio.2019.04.005. Epub 2019 Apr 17. PMID: 31002851.
120. Hosseinpour S, Tunér J, Fekrazad R. Photobiomodulation in Oral Surgery: A Review. Photobiomodul Photomed Laser Surg. 2019 Dec;37(12):814-825. doi: 10.1089/photob.2019.4712. Epub 2019 Nov 21. PMID: 31750798.
121. Huang Z, Chen J, Ma J, Shen B, Pei F, Kraus VB. Effectiveness of low-level laser therapy in patients with knee osteoarthritis: a systematic review and meta-analysis. Osteoarthritis Cartilage. 2015 Sep;23(9):1437-1444. doi: 10.1016/j.joca.2015.04.005. Epub 2015 Apr 23. PMID: 25914044; PMCID: PMC4814167.
122. Huang Z, Ma J, Chen J, Shen B, Pei F, Kraus VB. The effectiveness of low-level laser therapy for nonspecific chronic low back pain: a systematic review and meta-analysis. Arthritis Res Ther. 2015 Dec 15;17:360. doi: 10.1186/s13075-015-0882-0. PMID: 26667480; PMCID: PMC4704537.
123. Huang T, Wang Z, Li J. Efficiency of photobiomodulation on accelerating the tooth movement in the alignment phase of orthodontic treatment-A systematic review and meta-analysis. Heliyon. 2023;9(2):e13220. Published 2023 Jan 24. doi:10.1016/j.heliyon.2023.e13220
124. Huisstede BM, Hoogvliet P, Franke TP, Randsdorp MS, Koes BW. Carpal Tunnel Syndrome: Effectiveness of Physical Therapy and Electrophysical Modalities. An Updated Systematic Review of Randomized Controlled Trials. Arch Phys Med Rehabil. 2018 Aug;99(8):1623-1634.e23. doi: 10.1016/j.apmr.2017.08.482. Epub 2017 Sep 20. PMID: 28942118.
125. Ibarra AMC, Biasotto-Gonzalez DA, Kohatsu EYI, de Oliveira SSI, Bussadori SK, Tanganeli JPC. Photobiomodulation on trigeminal neuralgia: systematic review. Lasers Med Sci. 2021;36(4):715-722. doi:10.1007/s10103-020-03198-6
126. Imani MM, Golshah A, Safari-Faramani R, Sadeghi M. Effect of Low-level Laser Therapy on Orthodontic Movement of Human Canine: a Systematic Review and Meta-analysis of Randomized Clinical Trials. Acta Inform Med. 2018 Jun;26(2):139-143. doi: 10.5455/aim.2018.26.139-143. PMID: 30061788; PMCID: PMC6029894.
127. Jajarm HA, Asadi R, Bardideh E, Shafaee H, Khazaei Y, Emadzadeh M. The effects of photodynamic and low-level laser therapy for treatment of oral lichen planus-A systematic review and meta-analysis. Photodiagnosis Photodyn Ther. 2018;23:254-260. doi:10.1016/j.pdpdt.2018.07.001
128. Jang H, Lee H. Meta-analysis of pain relief effects by laser irradiation on joint areas. Photomed Laser Surg. 2012 Aug;30(8):405-17. doi: 10.1089/pho.2012.3240. Epub 2012 Jun 29. PMID: 22747309; PMCID: PMC3412059.
129. Javaherian M, Attarbashi Moghaddam B, Bashardoust Tajali S, Dabbaghipour N. Efficacy of low-level laser therapy on management of Bell's palsy: a systematic review. Lasers Med Sci. 2020 Aug;35(6):1245-1252. doi: 10.1007/s10103-020-02996-2. Epub 2020 Apr 21. PMID: 32318918.
130. Jedliński M, Romeo U, Del Vecchio A, Palaia G, Galluccio G. Comparison of the Effects of Photobiomodulation with Different Lasers on Orthodontic Movement and Reduction of the Treatment Time with Fixed Appliances in Novel Scientific Reports: A Systematic Review with Meta-Analysis. Photobiomodul Photomed Laser Surg. 2020 Aug;38(8):455-465. doi: 10.1089/photob.2019.4779. Epub 2020 Jul 15. PMID: 32678697.
131. Ji Q, Yan S, Ding J, et al. Photobiomodulation improves depression symptoms: a systematic review and meta-analysis of randomized controlled trials. Front Psychiatry. 2024;14:1267415. Published 2024 Jan 31. doi:10.3389/fpsyt.2023.1267415
132. Jiménez A, Carrick FR, Hoffman N, Jemni M. The Impact of Low-Level Laser Therapy on Spasticity in Children with Spastic Cerebral Palsy: A Systematic Review. Brain Sci. 2024;14(12):1179. Published 2024 Nov 25. doi:10.3390/brainsci14121179
133. John H, Manoloudakis N, Stephen Sinclair J. A systematic review of the use of lasers for the treatment of hidradenitis suppurativa. J Plast Reconstr Aesthet Surg. 2016 Oct;69(10):1374-81. doi: 10.1016/j.bjps.2016.05.029. Epub 2016 Jun 27. PMID: 27496291.
134. Joseph B, Mauramo M, Sorsa T, Anil S, Waltimo T. LED-based low-level light therapy for oral mucositis in cancer patients: a systematic review and GRADE analysis. Oral Surg Oral Med Oral Pathol Oral Radiol. Published online April 22, 2025. doi:10.1016/j.oooo.2025.04.095
135. Kadhim-Saleh A, Maganti H, Ghert M, Singh S, Farrokhyar F. Is low-level laser therapy in relieving neck pain effective? Systematic review and meta-analysis. Rheumatol Int. 2013 Oct;33(10):2493-501. doi: 10.1007/s00296-013-2742-z. Epub 2013 Apr 12. PMID: 23579335.
136. Karlsson MR, Diogo Löfgren CI, Jansson HM. The effect of laser therapy as an adjunct to non-surgical periodontal treatment in subjects with chronic periodontitis: a systematic review. J Periodontol. 2008 Nov;79(11):2021-8. doi: 10.1902/jop.2008.080197. PMID: 18980508.
137. Kauark-Fontes E, Rodrigues-Oliveira L, Epstein JB, et al. Cost-effectiveness of photobiomodulation therapy for the prevention and management of cancer treatment toxicities: a systematic review. Support Care Cancer. 2021;29(6):2875-2884. doi:10.1007/s00520-020-05949-1
138. Kechichian E, Jabbour S, El Hachem L, Tomb R, Helou J. Light and Laser Treatments for Keratosis Pilaris: A Systematic Review. Dermatol Surg. 2020 Nov;46(11):1397-1402. doi: 10.1097/DSS.0000000000002441. PMID: 32804891.
139. Khalil M, Hamadah O, Saifo M, et al. Effect of Photobiomodulation on Salivary Cytokines in Head and Neck Cancer Patients with Oral Mucositis: A Systematic Review. J Clin Med. 2024;13(10):2822. Published 2024 May 10. doi:10.3390/jcm13102822
140. Khan MK, Gurunathan D, Pandiyan R, Kumar A. Effectiveness of laser photobiomodulation therapy in pulpotomy of primary teeth: A systematic review. J Indian Soc Pedod Prev Dent. 2025;43(1):28-43. doi:10.4103/jisppd.jisppd_482_24
141. Khemiss M, Dammak N, Lajili O, Yacoub S, Ben Khelifa M. Efficacy of laser therapy on primary burning mouth syndrome: a systematic review. J Oral Facial Pain Headache. 2024;38(1):17-31. doi:10.22514/jofph.2024.003
142. Lai PS, Fierro C, Bravo L, Perez-Flores A. Benefits of Using Low-level Laser Therapy in the Rapid Maxillary Expansion: A Systematic Review. Int J Clin Pediatr Dent. 2021;14(Suppl 1):S101-S106. doi: 10.5005/jp-journals-10005-1966. PMID: 35082475; PMCID: PMC8754280.
143. Lauxen AC, Machado DR, Pereira DS, et al. Photobiomodulation in carpal tunnel syndrome with pain, strength, and functionality analysis: a systematic review and meta-analysis. Lasers Med Sci. 2025;40(1):12. Published 2025 Jan 8. doi:10.1007/s10103-024-04276-9
144. Leal-Junior EC, Vanin AA, Miranda EF, de Carvalho Pde T, Dal Corso S, Bjordal JM. Effect of phototherapy (low-level laser therapy and light-emitting diode therapy) on exercise performance and markers of exercise recovery: a systematic review with meta-analysis. Lasers Med Sci. 2015 Feb;30(2):925-39. doi: 10.1007/s10103-013-1465-4. Epub 2013 Nov 19. PMID: 24249354.
145. Li FJ, Zhang JY, Zeng XT, Guo Y. Low-level laser therapy for orthodontic pain: a systematic review. Lasers Med Sci. 2015 Aug;30(6):1789-803. doi: 10.1007/s10103-014-1661-x. Epub 2014 Sep 26. PMID: 25258106.
146. Li ZJ, Wang Y, Zhang HF, Ma XL, Tian P, Huang Y. Effectiveness of low-level laser on carpal tunnel syndrome: A meta-analysis of previously reported randomized trials. Medicine (Baltimore). 2016 Aug;95(31):e4424. doi: 10.1097/MD.0000000000004424. PMID: 27495063; PMCID: PMC4979817.
147. Li S, Wang C, Wang B, Liu L, Tang L, Liu D, Yang G, Zhang L. Efficacy of low-level light therapy for treatment of diabetic foot ulcer: A systematic review and meta-analysis of randomized controlled trials. Diabetes Res Clin Pract. 2018 Sep;143:215-224. doi: 10.1016/j.diabres.2018.07.014. Epub 2018 Jul 23. PMID: 30009935.
148. Lima MTE, E Lima JG, de Andrade MF, Bergmann A. Low-level laser therapy in secondary lymphedema after breast cancer: systematic review. Lasers Med Sci. 2014 May;29(3):1289-95. doi: 10.1007/s10103-012-1240-y. Epub 2012 Nov 29. PMID: 23192573.
149. Lin HW, Chen HC, Lin LF, Tam KW, Kuan YC. Laser therapy for Bell's palsy: a systematic review and meta-analysis of randomized trials. Lasers Med Sci. 2024;39(1):282. Published 2024 Nov 15. doi:10.1007/s10103-024-04237-2
150. Lin YT, Tung KM, Chiou JF, Chen YC, Hou WH. Effects of photobiomodulation therapy for acute radiation dermatitis in patients with cancer: A systematic review and meta‑analysis of real-world evidence. Radiother Oncol. 2025;202:110589. doi:10.1016/j.radonc.2024.110589
151. Liu KH, Liu D, Chen YT, Chin SY. Comparative effectiveness of low-level laser therapy for adult androgenic alopecia: a system review and meta-analysis of randomized controlled trials. Lasers Med Sci. 2019 Aug;34(6):1063-1069. doi: 10.1007/s10103-019-02723-6. Epub 2019 Jan 31. PMID: 30706177.
152. Louzeiro GC, Teixeira DDS, Cherubini K, de Figueiredo MAZ, Salum FG. Does laser photobiomodulation prevent hyposalivation in patients undergoing head and neck radiotherapy? A systematic review and meta-analysis of controlled trials. Crit Rev Oncol Hematol. 2020;156:103115. doi:10.1016/j.critrevonc.2020.103115
153. Lu C, Mo L, Li X, et al. Effects of low-level light therapy on pain and related lesions in patients with oral lichen planus: A systematic review and meta-analysis. J Evid Based Dent Pract. 2025;25(2):102126. doi:10.1016/j.jebdp.2025.102126
154. Luo WT, Lee CJ, Tam KW, Huang TW. Effects of Low-Level Laser Therapy on Muscular Performance and Soreness Recovery in Athletes: A Meta-analysis of Randomized Controlled Trials. Sports Health. 2022;14(5):687-693. doi:10.1177/19417381211039766
155. Luo Z, He Y, Wu H, et al. Efficacy of laser adjuvant therapy in the management of post-operative endodontic pain: A systematic review and meta-analysis. Int Endod J. 2024;57(12):1700-1716. doi:10.1111/iej.14140
156. Machado AC, Viana ÍEL, Farias-Neto AM, Braga MM, de Paula Eduardo C, de Freitas PM, et al. Is photobiomodulation (PBM) effective for the treatment of dentin hypersensitivity? A systematic review. Lasers Med Sci. 2018 May;33(4):745-753. doi: 10.1007/s10103-017-2403-7. Epub 2017 Dec 5. PMID: 29204915.
157. Machado RS, Viana S, Sbruzzi G. Low-level laser therapy in the treatment of pressure ulcers: systematic review. Lasers Med Sci. 2017;32(4):937-944. doi:10.1007/s10103-017-2150-9.
158. Machado AF, Micheletti JK, Lopes JSS, et al. Phototherapy on Management of Creatine Kinase Activity in General Versus Localized Exercise: A Systematic Review and Meta-Analysis. Clin J Sport Med. 2020;30(3):267-274. doi:10.1097/JSM.0000000000000606.
159. Mahintach T, Hascoet E, Cloitre A, Chaux AG. Impact of photobiomodulation in alveolar ridge preservation and implant stability after a dental extraction: a systematic review. *Lasers Med Sci*. 2024;39(1):264. Published 2024 Oct 26. doi:10.1007/s10103-024-04215-8
160. Mahmood D, Ahmad A, Sharif F, Arslan SA. Clinical application of low-level laser therapy (Photo-biomodulation therapy) in the management of breast cancer-related lymphedema: a systematic review. BMC Cancer. 2022;22(1):937. Published 2022 Aug 30. doi:10.1186/s12885-022-10021-8.
161. Mahuli SA, Rai A, Shree P, Ul Haque Z, Mahuli AV. Efficacy of photobiomodulation in the management of oral Lichen Planus in comparison to topical corticosteroids: Systematic review, meta-analysis, and GRADE-based assessment of certainty of evidence. J Stomatol Oral Maxillofac Surg. 2024;125(5S2):101798. doi:10.1016/j.jormas.2024.101798.
162. Maia ML de M, Bonjardim LR, Quintans J de SS, Ribeiro MAG, Maia LGM, Conti PCR. Effect of low-level laser therapy on pain levels in patients with temporomandibular disorders: a systematic review. J Appl Oral Sci. 2012 Nov; 20(6):594–602. Available from: <https://doi.org/10.1590/S1678-77572012000600002>.
163. Malik S, Sharma S, Dutta N, Khurana D, Sharma RK, Sharma S. Effect of low-level laser therapy plus exercise therapy on pain, range of motion, muscle strength, and function in knee osteoarthritis - a systematic review and meta-analysis. Somatosens Mot Res. 2023;40(1):8-24. doi:10.1080/08990220.2022.2157387.
164. Martimbianco ALC, Ferreira RES, Latorraca COC, Bussadori SK, Pacheco RL, Riera R. Photobiomodulation with low-level laser therapy for treating Achilles tendinopathy: a systematic review and meta-analysis. Clin Rehabil. 2020;34(6):713-722. doi:10.1177/0269215520912820.
165. Matos AL, Silva PU, Paranhos LR, Santana IT, Matos FR. Efficacy of the laser at low intensity on primary burning oral syndrome: a systematic review. Med Oral Patol Oral Cir Bucal. 2021;26(2):e216-e225. Published 2021 Mar 1. doi:10.4317/medoral.24144.
166. Máximo CFGP, Coêlho JF, Benevides SD, Alves GÂDS. Effects of low-level laser photobiomodulation on the masticatory function and mandibular movements in adults with temporomandibular disorder: a systematic review with meta-analysis. Fotobiomodulação com laser de baixa potência na função mastigatória e nos movimentos mandibulares em adultos com disfunção temporomandibular: revisão sistemática com metanálise. Codas. 2022;34(3):e20210138. Published 2022 Jan 31. doi:10.1590/2317-1782/20212021138.
167. Melis M, Di Giosia M, Zawawi KH. Low level laser therapy for the treatment of temporomandibular disorders: a systematic review of the literature. Cranio. 2012 Oct;30(4):304-12. doi: 10.1179/crn.2012.045. PMID: 23156972.
168. Meneses-Santos D, Costa MDMA, Inocêncio GSG, Almeida AC, Vieira WA, Lima IFP, et al. Effects of low-level laser therapy on reducing pain, edema, and trismus after orthognathic surgery: a systematic review. Lasers Med Sci. 2022 Apr;37(3):1471-1485. doi: 10.1007/s10103-021-03467-y. Epub 2021 Nov 17. PMID: 34791563.
169. Mesquita CM, Oliveira MB, Costa MDMA, et al. Effect of photobiomodulation therapy on pain perception during anesthetic puncture of dental local anesthesia: A systematic review. Clinics (Sao Paulo). 2024;79:100322. Published 2024 Mar 13. doi:10.1016/j.clinsp.2023.100322.
170. Michelogiannakis D, Jabr L, Barmak AB, Rossouw PE, Kotsailidi EA, Javed F. Influence of low-level-laser therapy on the stability of orthodontic mini-screw implants. A systematic review and meta-analysis. Eur J Orthod. 2022;44(1):11-21. doi:10.1093/ejo/cjab016.
171. Migliorati C, Hewson I, Lalla RV, Antunes HS, Estilo CL, Hodgson B, et al. Mucositis Study Group of the Multinational Association of Supportive Care in Cancer/International Society of Oral Oncology (MASCC/ISOO). Systematic review of laser and other light therapy for the management of oral mucositis in cancer patients. Support Care Cancer. 2013 Jan;21(1):333-41. doi: 10.1007/s00520-012-1605-6. Epub 2012 Sep 22. PMID: 23001179.
172. Mikami R, Mizutani K, Sasaki Y, Iwata T, Aoki A. Patient-reported outcomes of laser-assisted pain control following non-surgical and surgical periodontal therapy: A systematic review and meta-analysis. PLoS One. 2020 Sep 17;15(9):e0238659. doi: 10.1371/journal.pone.0238659. PMID: 32941479; PMCID: PMC7498060.
173. Minervini G, Franco R, Martelli M, et al. Low-level laser treatment's ability to reduce dry socket pain. Acta Odontol Scand. 2024;83:631-641. Published 2024 Nov 12. doi:10.2340/aos.v83.42261.
174. Mirzaei A, Saberi-Demneh A, Gutknecht N, Ramezani G. The effect of low-level laser radiation on improving inferior alveolar nerve damage after sagittal split osteotomy: a systematic review. Lasers Med Sci. 2019 Jul;34(5):865-872. doi: 10.1007/s10103-019-02718-3. Epub 2019 Jan 19. PMID: 30661183.
175. Mokeem S. Efficacy of adjunctive low-level laser therapy in the treatment of aggressive periodontitis: A systematic review. J Investig Clin Dent. 2018 Nov;9(4):e12361. doi: 10.1111/jicd.12361. Epub 2018 Sep 9. PMID: 30198204.
176. Munguia FM, Jang J, Salem M, Clark GT, Enciso R. Efficacy of Low-Level Laser Therapy in the Treatment of Temporomandibular Myofascial Pain: A Systematic Review and Meta-Analysis. J Oral Facial Pain Headache. 2018 Summer;32(3):287–297. doi: 10.11607/ofph.2032. Epub 2018 Apr 25. PMID: 29697718.
177. Najem I, Chen H. Use of low-level laser therapy in treatment of the androgenic alopecia, the first systematic review. J Cosmet Laser Ther. 2018;20(4):252-257. doi:10.1080/14764172.2017.1400174.
178. Nampo FK, Cavalheri V, Dos Santos Soares F, de Paula Ramos S, Camargo EA. Low-level phototherapy to improve exercise capacity and muscle performance: a systematic review and meta-analysis. Lasers Med Sci. 2016 Dec;31(9):1957-1970. doi: 10.1007/s10103-016-1977-9. Epub 2016 Jun 7. PMID: 27272746.
179. Nampo FK, Cavalheri V, Ramos Sde P, Camargo EA. Effect of low-level phototherapy on delayed onset muscle soreness: a systematic review and meta-analysis. Lasers Med Sci. 2016 Jan;31(1):165-77. doi: 10.1007/s10103-015-1832-4. Epub 2015 Nov 12. PMID: 26563953.
180. Naterstad IF, Joensen J, Bjordal JM, Couppé C, Lopes-Martins RAB, Stausholm MB. Efficacy of low-level laser therapy in patients with lower extremity tendinopathy or plantar fasciitis: systematic review and meta-analysis of randomised controlled trials. BMJ Open. 2022;12(9):e059479. Published 2022 Sep 28. doi:10.1136/bmjopen-2021-059479.
181. Navarro-Fernández G, Gil-Martínez A, Diaz-Saez MC, et al. Effectiveness of Physical Therapy in Orthognathic Surgery Patients: A Systematic Review of Randomized Controlled Trials. J Funct Morphol Kinesiol. 2023;8(1):17. Published 2023 Jan 30. doi:10.3390/jfmk8010017.
182. Nayyer N, Tripathi T, Ganesh G, Rai P. Impact of photobiomodulation on external root resorption during orthodontic tooth movement in humans - A systematic review and meta-analysis. J Oral Biol Craniofac Res. 2022;12(4):469-480. doi:10.1016/j.jobcr.2022.05.014.
183. Nogueira AC Jr, Júnior Mde J. The effects of laser treatment in tendinopathy: a systematic review. Acta Ortop Bras. 2015 Jan-Feb;23(1):47-9. doi: 10.1590/1413-78522015230100513. PMID: 26327796; PMCID: PMC4544521.
184. Nunes LP, Nunes GP, Ferrisse TM, et al. A systematic review and meta-analysis on the effects of phototherapy on postoperative pain in conventional endodontic reintervention. Clin Oral Investig. 2024;28(4):232. Published 2024 Apr 1. doi:10.1007/s00784-024-05623-4.
185. Oberoi S, Zamperlini-Netto G, Beyene J, Treister NS, Sung L. Effect of prophylactic low level laser therapy on oral mucositis: a systematic review and meta-analysis. PLoS One. 2014 Sep 8;9(9):e107418. doi: 10.1371/journal.pone.0107418. PMID: 25198431; PMCID: PMC4157876.
186. Okuhara MR, Trevisani VFM, Macedo CR. Effects of Photobiomodulation on Burning Mouth Syndrome: A Systematic Review and Meta-Analysis. J Oral Rehabil. 2025;52(4):540-553. doi:10.1111/joor.13931.
187. Oliveira S, Andrade R, Valente C, et al. Effectiveness of Photobiomodulation in Reducing Pain and Disability in Patients With Knee Osteoarthritis: A Systematic Review With Meta-Analysis. Phys Ther. 2024;104(8):pzae073. doi:10.1093/ptj/pzae073.
188. Olszewska A, Wolny M, Kensy J, Kotela A, Czajka-Jakubowska A, Matys J. Photobiomodulation Therapy for Neurosensory Disturbances in Orthognathic Surgery Patients: A Systematic Review. Life (Basel). 2025;15(1):111. Published 2025 Jan 16. doi:10.3390/life15010111.
189. Omar MT, Shaheen AA, Zafar H. A systematic review of the effect of low-level laser therapy in the management of breast cancer-related lymphedema. Support Care Cancer. 2012 Nov;20(11):2977-84. doi: 10.1007/s00520-012-1546-0. Epub 2012 Aug 9. PMID: 22875413.
190. Pacheco JA, Molena KF, Martins CROG, Corona SAM, Borsatto MC. Photobiomodulation (PBMT) and antimicrobial photodynamic therapy (aPDT) in oral manifestations of patients infected by Sars-CoV-2: systematic review and meta-analysis. Bull Natl Res Cent. 2022;46(1):140. doi: 10.1186/s42269-022-00830-z. Epub 2022 May 16. PMID: 35601476; PMCID: PMC9108688.
191. Paglioni MP, Alves CGB, Fontes EK, Lopes MA, Ribeiro ACP, Brandão TB, Migliorati CA, Santos-Silva AR. Is photobiomodulation therapy effective in reducing pain caused by toxicities related to head and neck cancer treatment? A systematic review. Support Care Cancer. 2019 Nov;27(11):4043-4054. doi: 10.1007/s00520-019-04939-2. Epub 2019 Jul 1. PMID: 31264186.
192. Parker S, Cronshaw M, Anagnostaki E, Bordin-Aykroyd SR, Lynch E. Systematic Review of Delivery Parameters Used in Dental Photobiomodulation Therapy. Photobiomodul Photomed Laser Surg. 2019 Dec;37(12):784-797. doi: 10.1089/photob.2019.4694. Epub 2019 Oct 1. PMID: 31573388.
193. Parker S, Anagnostaki E, Mylona V, Cronshaw M, Lynch E, Grootveld M. Systematic Review of Post-Surgical Laser-Assisted Oral Soft Tissue Outcomes Using Surgical Wavelengths Outside the 650-1350 nm Optical Window. Photobiomodul Photomed Laser Surg. 2020 Oct;38(10):591-606. doi: 10.1089/photob.2020.4847. PMID: 33026973.
194. Parra-Rojas S, Velázquez-Cayón RT, Ciortan-Pop ME, Martins MD, Cassol Spanemberg J. Preventive Photobiomodulation for Chemotherapy-Induced Oral Mucositis: A Systematic Review of Randomized Clinical Trials. Biomedicines. 2025; 13(2):268. https://doi.org/10.3390/biomedicines13020268.
195. Pavlić V. [The effects of low-level laser therapy on xerostomia (mouth dryness)]. Med Pregl. 2012 May-Jun;65(5-6):247-50. Serbian. doi: 10.2298/mpns1206247p. PMID: 22730712.
196. Penberthy WT, Vorwaller CE. Utilization of the 1064 nm Wavelength in Photobiomodulation: A Systematic Review and Meta-Analysis. J Lasers Med Sci. 2021 Dec 28;12:e86. doi: 10.34172/jlms.2021.86. PMID: 35155171; PMCID: PMC8837867.
197. Peng J, Shi Y, Wang J, Wang F, Dan H, Xu H, Zeng X. Low-level laser therapy in the prevention and treatment of oral mucositis: a systematic review and meta-analysis. Oral Surg Oral Med Oral Pathol Oral Radiol. 2020 Oct;130(4):387-397.e9. doi: 10.1016/j.oooo.2020.05.014. Epub 2020 Jun 5. PMID: 32624448.
198. Peralta-Mamani M, da Silva BM, da Silva Pinto AC, Rubira-Bullen IRF, Honório HM, Rubira CMF, da Silva Santos PS. Low-level laser therapy dosimetry most used for oral mucositis due to radiotherapy for head and neck cancer: a systematic review and meta-analysis. Crit Rev Oncol Hematol. 2019 Jun;138:14-23. doi: 10.1016/j.critrevonc.2019.03.009. Epub 2019 Mar 31. PMID: 31092370.
199. Petrucci A, Sgolastra F, Gatto R, Mattei A, Monaco A. Effectiveness of low-level laser therapy in temporomandibular disorders: a systematic review and meta-analysis. J Orofac Pain. 2011 Fall;25(4):298-307. PMID: 22247925.
200. Petz FFC, Félix JVC, Roehrs H, Pott FS, Stocco JGD, Marcos RL, Meier MJ. Effect of Photobiomodulation on Repairing Pressure Ulcers in Adult and Elderly Patients: A Systematic Review. Photochem Photobiol. 2020 Jan;96(1):191-199. doi: 10.1111/php.13162. Epub 2019 Nov 10. PMID: 31550398.
201. Qadri T, Javed F, Johannsen G, Gustafsson A. Role of diode lasers (800-980 nm) as adjuncts to scaling and root planing in the treatment of chronic periodontitis: a systematic review. Photomed Laser Surg. 2015 Nov;33(11):568-75. doi: 10.1089/pho.2015.3914. Epub 2015 Oct 5. PMID: 26436596.
202. Radithia D, Mahdani FY, Bakti RK, et al. Effectiveness of low-level laser therapy in reducing pain score and healing time of recurrent aphthous stomatitis: a systematic review and meta-analysis. Syst Rev. 2024;13(1):192. Published 2024 Jul 22. doi:10.1186/s13643-024-02595-0.
203. Rajai Firouzabadi S, Mohammadi I, Aarabi A, Sadraei S. Low-Level Laser Therapy for Allergic Rhinitis: A Systematic Review and Meta-Analysis. Int Arch Allergy Immunol. 2024;185(9):871-883. doi:10.1159/000538049.
204. Ramezani F, Razmgir M, Tanha K, Nasirinezhad F, Neshastehriz A, Bahrami-Ahmadi A, Hamblin MR, Janzadeh A. Photobiomodulation for spinal cord injury: A systematic review and meta-analysis. Physiol Behav. 2020 Oct 1;224:112977. doi: 10.1016/j.physbeh.2020.112977. Epub 2020 Jun 3. PMID: 32504695.
205. Rani S, Dhawan P, Kruthiventi H. Clinical efficacy of photobiomodulation therapy in dental implant stability and crestal bone loss in implants placed in healed sites: a systematic review and meta-analysis of randomized clinical trials. Lasers Med Sci. 2025;40(1):40. Published 2025 Jan 24. doi:10.1007/s10103-024-04258-x.
206. Rayegani SM, Raeissadat SA, Heidari S, Moradi-Joo M. Safety and Effectiveness of Low-Level Laser Therapy in Patients With Knee Osteoarthritis: A Systematic Review and Meta-analysis. J Lasers Med Sci. 2017 Summer;8(Suppl 1):S12-S19. doi: 10.15171/jlms.2017.s3. Epub 2017 Aug 29. PMID: 29071029; PMCID: PMC5642172.
207. Rayegani SM, Moradi-Joo M, Raeissadat SA, Bahrami MH, Seyed-Nezhad M, Heidari S. Effectiveness of Low-Level Laser Therapy compared to Ultrasound in Patients With Carpal Tunnel Syndrome: A Systematic Review and Meta-analysis. J Lasers Med Sci. 2019 Fall;10(Suppl 1):S82-S89. doi: 10.15171/jlms.2019.S15. Epub 2019 Dec 1. PMID: 32021679; PMCID: PMC6983862.
208. Redman MG, Harris K, Phillips BS. Low-level laser therapy for oral mucositis in children with cancer. Arch Dis Child. 2022 Feb;107(2):128-133. doi: 10.1136/archdischild-2020-321216. Epub 2021 Jul 6. PMID: 34230010.
209. Reis CHB, Buchaim DV, Ortiz AC, Fideles SOM, Dias JA, Miglino MA, Teixeira DB, Pereira ESBM, da Cunha MR, Buchaim RL. Application of Fibrin Associated with Photobiomodulation as a Promising Strategy to Improve Regeneration in Tissue Engineering: A Systematic Review. Polymers (Basel). 2022 Aug 2;14(15):3150. doi: 10.3390/polym14153150. PMID: 35956667; PMCID: PMC9370794.
210. Ren C, McGrath C, Yang Y. The effectiveness of low-level diode laser therapy on orthodontic pain management: a systematic review and meta-analysis. Lasers Med Sci. 2015 Sep;30(7):1881-93. doi: 10.1007/s10103-015-1743-4. Epub 2015 Mar 24. PMID: 25800534; PMCID: PMC4562996.
211. Ren C, McGrath C, Jin L, Zhang C, Yang Y. The effectiveness of low-level laser therapy as an adjunct to non-surgical periodontal treatment: a meta-analysis. J Periodontal Res. 2017 Feb;52(1):8-20. doi: 10.1111/jre.12361. Epub 2016 Mar 2. PMID: 26932392; PMCID: PMC5297978.
212. Ribeiro LN, de Vasconcelos Carvalho M, de Oliveira Limirio JPJ, do Egito Vasconcelos BC, Moraes SLD, Pellizzer EP. Impact of low-level laser therapy on the quality of life of patients with xerostomia undergoing head and neck radiotherapy: a systematic review. Support Care Cancer. 2024;32(2):118. Published 2024 Jan 20. doi:10.1007/s00520-024-08325-5.
213. Ricci NA, Dias CN, Driusso P. A utilização dos recursos eletrotermofototerapêuticos no tratamento da síndrome da fibromialgia: uma revisão sistemática [The use of electrothermal and phototherapeutic methods for the treatment of fibromyalgia syndrome: a systematic review]. Rev Bras Fisioter. 2010 Jan-Feb;14(1):1-9. Portuguese. PMID: 20414555.
214. Rocha SR, da Costa Ferreira SA, Ramalho A, Conceição Gouveia Santos VL, Cristina Nogueira P. Photobiomodulation Therapy in the Prevention and Treatment of Radiodermatitis in Breast Cancer Patients: Systematic Review. J Lasers Med Sci. 2022 Oct 2;13:e42. doi: 10.34172/jlms.2022.42. PMID: 36743146; PMCID: PMC9841382.
215. Ruiz Roca JA, López Jornet P, Gómez García FJ, Marcos Aroca P. Effect of Photobiomodulation on Atrophic-Erosive Clinical Forms of Oral Lichen Planus: A Systematic Review. Dent J (Basel). 2022 Nov 27;10(12):221. doi: 10.3390/dj10120221. PMID: 36547037; PMCID: PMC9776719.
216. Sadeghian A, Rohani B, Salehi-Marzijarani M, Fekrazad R. Radiographical impact of photobiomodulation therapy on bone regeneration in clinical studies: a systematic review. Lasers Med Sci. 2025;40(1):23. Published 2025 Jan 20. doi:10.1007/s10103-024-04244-3.
217. Saini RS, Kanji MA, Okshah A, et al. Comparative efficacy of photobiomodulation on osseointegration in dental implants: A systematic review and meta-analysis. Photodiagnosis Photodyn Ther. 2024;48:104256. doi:10.1016/j.pdpdt.2024.104256.
218. Salehpour F, Majdi A, Pazhuhi M, Ghasemi F, Khademi M, Pashazadeh F, Hamblin MR, Cassano P. Transcranial Photobiomodulation Improves Cognitive Performance in Young Healthy Adults: A Systematic Review and Meta-Analysis. Photobiomodul Photomed Laser Surg. 2019 Oct;37(10):635-643. doi: 10.1089/photob.2019.4673. Epub 2019 Sep 24. PMID: 31549906; PMCID: PMC6818490.
219. Saneja R, Bhattacharjee B, Bhatnagar A, Kumar PGN, Verma A. Efficacy of different lasers of various wavelengths in treatment of peri-implantitis and peri-implant mucositis: A systematic review and meta-analysis. J Indian Prosthodont Soc. 2020 Oct-Dec;20(4):353-362. doi: 10.4103/jips.jips_144_20. Epub 2020 Oct 8. PMID: 33487962; PMCID: PMC7814680.
220. Salajegheh A, Yahyaabadi FY, Yazdi F. Low level laser therapy and rheumatoid arthritis: a systematic review and meta-analysis study. Eur J Transl Myol. 2024;34(4):13107. Published 2024 Nov 21. doi:10.4081/ejtm.2024.13107.
221. Sales PVA, Godói IPD, Brito GAC, Leitão RC, Araújo AA, Medeiros CACX. Mechanisms of photobiomodulation therapy in treating and preventing antineoplastic-induced oral mucositis: a systematic review. Acta Cir Bras. 2025;40:e403125. Published 2025 Mar 31. doi:10.1590/acb403125.
222. Santinoni CD, Oliveira HF, Batista VE, Lemos CA, Verri FR. Influence of low-level laser therapy on the healing of human bone maxillofacial defects: A systematic review. J Photochem Photobiol B. 2017 Apr;169:83-89. doi: 10.1016/j.jphotobiol.2017.03.004. Epub 2017 Mar 7. PMID: 28292696.
223. Santos CMD, Rocha RBD, Hazime FA, Cardoso VS. A Systematic Review and Meta-Analysis of the Effects of Low-Level Laser Therapy in the Treatment of Diabetic Foot Ulcers. Int J Low Extrem Wounds. 2021 Sep;20(3):198-207. doi: 10.1177/1534734620914439. Epub 2020 May 12. PMID: 32394760.
224. Seyyedi SA, Fini MB, Fekrazad R, Abbasian S, Abdollahi AA. Effect of photobiomodulation on postoperative endodontic pain: A systematic review of clinical trials. Dent Res J (Isfahan). 2024;21:7. Published 2024 Jan 25.
225. Shadid RM, Sadaqah NR, Othman SA. Does the Implant Surgical Technique Affect the Primary and/or Secondary Stability of Dental Implants? A Systematic Review. Int J Dent. 2014;2014:204838. doi: 10.1155/2014/204838. Epub 2014 Jul 7. PMID: 25126094; PMCID: PMC4121016.
226. Shafaee H, Bardideh E, Nazari MS, Asadi R, Shahidi B, Rangrazi A. The effects of photobiomodulation therapy for treatment of alveolar osteitis (Dry Socket): Systematic review and meta-analysis. Photodiagnosis Photodyn Ther. 2020 Dec;32:102000. doi: 10.1016/j.pdpdt.2020.102000. Epub 2020 Sep 9. PMID: 32919077.
227. ​​Shan Z, Ji J, McGrath C, Gu M, Yang Y. Effects of low-level light therapy on dentin hypersensitivity: a systematic review and meta-analysis. Clin Oral Investig. 2021;25(12):6571-6595. doi:10.1007/s00784-021-04183-1.
228. Shukla D, Muthusekhar MR. Efficacy of low-level laser therapy in temporomandibular disorders: A systematic review. Natl J Maxillofac Surg. 2016 Jan-Jun;7(1):62-66. doi: 10.4103/0975-5950.196127. PMID: 28163481; PMCID: PMC5242077.
229. Sims SE, Miller K, Elfar JC, Hammert WC. Non-surgical treatment of lateral epicondylitis: a systematic review of randomized controlled trials. Hand (N Y). 2014 Dec;9(4):419-46. doi: 10.1007/s11552-014-9642-x. PMID: 25414603; PMCID: PMC4235906.
230. Slot DE, Jorritsma KH, Cobb CM, Van der Weijden FA. The effect of the thermal diode laser (wavelength 808-980 nm) in non-surgical periodontal therapy: a systematic review and meta-analysis. J Clin Periodontol. 2014 Jul;41(7):681-92. doi: 10.1111/jcpe.12233. Epub 2014 Jun 2. PMID: 24460795.
231. Smoot B, Chiavola-Larson L, Lee J, Manibusan H, Allen DD. Effect of low-level laser therapy on pain and swelling in women with breast cancer-related lymphedema: a systematic review and meta-analysis. J Cancer Surviv. 2015 Jun;9(2):287-304. doi: 10.1007/s11764-014-0411-1. Epub 2014 Nov 29. PMID: 25432632.
232. Sobol M, Pniewski J. Efficacy of Repeated Low-Level Red Light (RLRL) Therapy in Managing Childhood Myopia: A Systematic Review and Meta-Analysis. J Clin Med. 2025; 14(1):83. https://doi.org/10.3390/jcm14010083.
233. Sobral AP, Sobral SS, Campos TM, Horliana AC, Fernandes KP, Bussadori SK, Motta LJ. Photobiomodulation and myofascial temporomandibular disorder: Systematic review and meta-analysis followed by cost-effectiveness analysis. J Clin Exp Dent. 2021 Jul 1;13(7):e724-e732. doi: 10.4317/jced.58084. PMID: 34306537; PMCID: PMC8291152.
234. Soh WK, Cheah KF, Veettil SK, Pandiar D, Nimbalkar S, Gopinath D. Photobiomodulation Therapy in the Management of Oral Lichen Planus: A Systematic Review and Meta-Analysis. Eur J Dent. 2024;18(4):976-986. doi:10.1055/s-0044-1782213
235. Stausholm MB, Naterstad IF, Joensen J, Lopes-Martins RÁB, Sæbø H, Lund H, Fersum KV, Bjordal JM. Efficacy of low-level laser therapy on pain and disability in knee osteoarthritis: systematic review and meta-analysis of randomised placebo-controlled trials. BMJ Open. 2019 Oct 28;9(10):e031142. doi: 10.1136/bmjopen-2019-031142. PMID: 31662383; PMCID: PMC6830679.
236. Sun Y, Li Z, Qi X, et al. Laser therapy for treating cleft lip or/and palate scarring-a systematic review and meta-analysis. Lasers Med Sci. 2024;39(1):160. Published 2024 Jun 20. doi:10.1007/s10103-024-04082-3
237. Sun W, Zhuang Z, Yang L, Zhou J, Zhang L, Yuan J. Effectiveness of photobiomodulation therapy in improving health indicators in obese patients: a systematic review and meta-analysis of RCTs. BMC Complement Med Ther. 2025;25(1):133. Published 2025 Apr 11. doi:10.1186/s12906-025-04874-2
238. Sussmilch-Leitch SP, Collins NJ, Bialocerkowski AE, Warden SJ, Crossley KM. Physical therapies for Achilles tendinopathy: systematic review and meta-analysis. J Foot Ankle Res. 2012 Jul 2;5(1):15. doi: 10.1186/1757-1146-5-15. PMID: 22747701; PMCID: PMC3537637.
239. Taberner-Vallverdú M, Nazir M, Sánchez-Garcés MÁ, Gay-Escoda C. Efficacy of different methods used for dry socket management: A systematic review. Med Oral Patol Oral Cir Bucal. 2015;20(5):e633-e639. Published 2015 Sep 1. doi:10.4317/medoral.20589
240. Taha N, Daoud H, Malik T, Shettysowkoor J, Rahman S. The Effects of Low-Level Laser Therapy on Wound Healing and Pain Management in Skin Wounds: A Systematic Review and Meta-Analysis. Cureus. 2024;16(10):e72542. Published 2024 Oct 28. doi:10.7759/cureus.72542
241. Talluri S, Palaparthi SM, Michelogiannakis D, Khan J. Efficacy of photobiomodulation in the management of tinnitus: A systematic review of randomized control trials. Eur Ann Otorhinolaryngol Head Neck Dis. 2022 Mar;139(2):83-90. doi: 10.1016/j.anorl.2020.10.013. Epub 2021 Mar 5. PMID: 33685826.
242. Tan K, Coster T, Mousa A, et al. Laser and Light-Based Therapies for Hirsutism Management in Women With Polycystic Ovarian Syndrome: A Systematic Review. JAMA Dermatol. 2024;160(7):746-757. doi:10.1001/jamadermatol.2024.0623
243. Tchanque-Fossuo CN, Ho D, Dahle SE, Koo E, Li CS, Isseroff RR, Jagdeo J. A systematic review of low-level light therapy for treatment of diabetic foot ulcer. Wound Repair Regen. 2016 Mar;24(2):418-26. doi: 10.1111/wrr.12399. Epub 2016 Mar 2. PMID: 26748691.
244. Tehrani MR, Nazary-Moghadam S, Zeinalzadeh A, Moradi A, Mehrad-Majd H, Sahebalam M. Efficacy of low-level laser therapy on pain, disability, pressure pain threshold, and range of motion in patients with myofascial neck pain syndrome: a systematic review and meta-analysis of randomized controlled trials. Lasers Med Sci. 2022 Dec;37(9):3333-3341. doi: 10.1007/s10103-022-03626-9. Epub 2022 Aug 13. PMID: 35962884.
245. Telles-Araujo GT, Cruz KMD, Preto KA, et al. Photobiomodulation in the management of persistent olfactory and gustatory dysfunction post-COVID-19: a systematic review. Lasers Med Sci. 2025;40(1):283. Published 2025 Jun 16. doi:10.1007/s10103-025-04534-4
246. Tengrungsun T, Mitrirattanakul S, Buranaprasertsuk P, Suddhasthir T. Is low level laser effective for the treatment of orofacial pain?: A systematic review. Cranio. 2012 Oct;30(4):280-5. doi: 10.1179/crn.2012.042. PMID: 23156969.
247. Tomazoni SS, Almeida MO, Bjordal JM, Stausholm MB, Machado CDSM, Leal-Junior ECP, Costa LOP. Photobiomodulation therapy does not decrease pain and disability in people with non-specific low back pain: a systematic review. J Physiother. 2020 Jul;66(3):155-165. doi: 10.1016/j.jphys.2020.06.010. Epub 2020 Jul 14. PMID: 32680739.
248. Toopalle SV, Yadav I, Gupta A, et al. Effect of Laser Therapy on Postoperative Pain and Endodontic Retreatment: A Systematic Review and Meta-Analysis. Int Dent J. 2024;74(2):335-342. doi:10.1016/j.identj.2023.10.012
249. Tournavitis A, Sandris E, Theocharidou A, Slini T, Kokoti M, Koidis P, Tortopidis D. Effectiveness of conservative therapeutic modalities for temporomandibular disorders-related pain: a systematic review. Acta Odontol Scand. 2023 May;81(4):286-297. doi: 10.1080/00016357.2022.2138967. Epub 2022 Nov 10. PMID: 36354093.
250. Tripodi N, Feehan J, Husaric M, Sidiroglou F, Apostolopoulos V. The effect of low-level red and near-infrared photobiomodulation on pain and function in tendinopathy: a systematic review and meta-analysis of randomized control trials. BMC Sports Sci Med Rehabil. 2021 Aug 14;13(1):91. doi: 10.1186/s13102-021-00306-z. PMID: 34391447; PMCID: PMC8364035.
251. Tumilty S, Munn J, McDonough S, Hurley DA, Basford JR, Baxter GD. Low level laser treatment of tendinopathy: a systematic review with meta-analysis. Photomed Laser Surg. 2010 Feb;28(1):3-16. doi: 10.1089/pho.2008.2470. PMID: 19708800.
252. Tunér J, Hosseinpour S, Fekrazad R. Photobiomodulation in Temporomandibular Disorders. Photobiomodul Photomed Laser Surg. 2019 Dec;37(12):826-836. doi: 10.1089/photob.2019.4705. Epub 2019 Nov 26. PMID: 31770071.
253. Ullah S, Umer MF, Chandran SP. Long-term effect of repeated low-level red light therapy on myopia control: A systematic review and meta-analysis. Eur J Ophthalmol. 2025;35(4):1432-1444. doi:10.1177/11206721251314541
254. Vale FA, Moreira MS, de Almeida FC, Ramalho KM. Low-level laser therapy in the treatment of recurrent aphthous ulcers: a systematic review. ScientificWorldJournal. 2015;2015:150412. doi: 10.1155/2015/150412. Epub 2015 Mar 23. PMID: 25879049; PMCID: PMC4386290.
255. Vande A, Sanyal PK, Nilesh K. Effectiveness of the photobiomodulation therapy using low-level laser around dental implants: A systematic review and meta-analysis. Dent Med Probl. 2022 Apr-Jun;59(2):281-289. doi: 10.17219/dmp/143242. PMID: 35686695.
256. Vanin AA, Verhagen E, Barboza SD, Costa LOP, Leal-Junior ECP. Photobiomodulation therapy for the improvement of muscular performance and reduction of muscular fatigue associated with exercise in healthy people: a systematic review and meta-analysis. Lasers Med Sci. 2018 Jan;33(1):181-214. doi: 10.1007/s10103-017-2368-6. Epub 2017 Oct 31. PMID: 29090398.
257. Vieceli AS, Martins JC, Hendler KG, Santos APT, das Neves LMS, Barbosa RI, Kuriki HU, Marcolino AM. Effectiveness of electrophysical agents for treating pressure injuries: a systematic review. Lasers Med Sci. 2022 Dec;37(9):3363-3377. doi: 10.1007/s10103-022-03648-3. Epub 2022 Oct 6. PMID: 36201144.
258. Vlassov VV, MacLehose HG. Low level laser therapy for treating tuberculosis. Cochrane Database Syst Rev. 2006 Apr 19;2006(2):CD003490. doi: 10.1002/14651858.CD003490.pub2. PMID: 16625582; PMCID: PMC6532747.
259. Vrijman C, van Drooge AM, Limpens J, Bos JD, van der Veen JP, Spuls PI, Wolkerstorfer A. Laser and intense pulsed light therapy for the treatment of hypertrophic scars: a systematic review. Br J Dermatol. 2011 Nov;165(5):934-42. doi: 10.1111/j.1365-2133.2011.10492.x. PMID: 21711337.
260. Wang W, Jiang W, Tang C, Zhang X, Xiang J. Clinical efficacy of low-level laser therapy in plantar fasciitis: A systematic review and meta-analysis. Medicine (Baltimore). 2019 Jan;98(3):e14088. doi: 10.1097/MD.0000000000014088. PMID: 30653125; PMCID: PMC6370152.
261. Weber JB, Camilotti RS, Ponte ME. Efficacy of laser therapy in the management of bisphosphonate-related osteonecrosis of the jaw (BRONJ): a systematic review. Lasers Med Sci. 2016 Aug;31(6):1261-72. doi: 10.1007/s10103-016-1929-4. Epub 2016 Mar 30. PMID: 27025860.
262. Wei S, Zhang G, Wu Q, Song T, Yin N, Wang Y. Efficacy and safety of phototherapies for upper lip scars in cleft lip patients: a systematic review and meta-analysis. Int J Surg. 2025;111(1):1407-1414. Published 2025 Jan 1. doi:10.1097/JS9.0000000000002055
263. Winters M, Eskes M, Weir A, Moen MH, Backx FJ, Bakker EW. Treatment of medial tibial stress syndrome: a systematic review. Sports Med. 2013 Dec;43(12):1315-33. doi: 10.1007/s40279-013-0087-0. PMID: 23979968.
264. Wu X, Zhu J, Zheng B, Liu J, Wu Z. Effectiveness of low-level gallium aluminium arsenide laser therapy for temporomandibular disorder with myofascial pain: A systemic review and meta-analysis. Medicine (Baltimore). 2021 Dec 30;100(52):e28015. doi: 10.1097/MD.0000000000028015. PMID: 34967349; PMCID: PMC8718212.
265. Xiaoting L, Yin T, Yangxi C. Interventions for pain during fixed orthodontic appliance therapy. A systematic review. Angle Orthod. 2010 Sep;80(5):925-32. doi: 10.2319/010410-10.1. PMID: 20578865; PMCID: PMC8939023.
266. Xu GZ, Jia J, Jin L, Li JH, Wang ZY, Cao DY. Low-Level Laser Therapy for Temporomandibular Disorders: A Systematic Review with Meta-Analysis. Pain Res Manag. 2018 May 10;2018:4230583. doi: 10.1155/2018/4230583. PMID: 29861802; PMCID: PMC5971344.
267. Yadav S, Sharma S, Chatterjee S, Sharma A, Thakur S. Effect of LASER therapy on plantar fasciitis pain: illuminating a promising treatment approach - a systematic review. Lasers Med Sci. 2025;40(1):18. Published 2025 Jan 15. doi:10.1007/s10103-025-04289-y
268. Yavagal CM, Matondkar SP, Yavagal PC. Efficacy of Laser Photobiomodulation in Accelerating Orthodontic Tooth Movement in Children: A Systematic Review with Meta-analysis. Int J Clin Pediatr Dent. 2021;14(Suppl 1):S94-S100. doi: 10.5005/jp-journals-10005-1964. PMID: 35082474; PMCID: PMC8754265.
269. Ye G, Ying Y, Shen B, Liu J, Lu J. Effect of intraoral photobiomodulation therapy on pain perception associated with local anaesthesia infiltration: a systematic review and meta-analysis of randomized controlled trials. Int J Oral Maxillofac Surg. 2025;54(1):82-92. doi:10.1016/j.ijom.2024.10.005
270. Yeh SW, Hong CH, Shih MC, Tam KW, Huang YH, Kuan YC. Low-Level Laser Therapy for Fibromyalgia: A Systematic Review and Meta-Analysis. Pain Physician. 2019 May;22(3):241-254. PMID: 31151332.
271. Youssef MA, Shehata AR, Adly AM, et al. Efficacy of Repeated Low-Level Red Light (RLRL) therapy on myopia outcomes in children: a systematic review and meta-analysis. BMC Ophthalmol. 2024;24(1):78. Published 2024 Feb 20. doi:10.1186/s12886-024-03337-5
272. Zadik Y, Arany PR, Fregnani ER, Bossi P, Antunes HS, Bensadoun RJ, Gueiros LA, Majorana A, Nair RG, Ranna V, Tissing WJE, Vaddi A, Lubart R, Migliorati CA, Lalla RV, Cheng KKF, Elad S; Mucositis Study Group of the Multinational Association of Supportive Care in Cancer/International Society of Oral Oncology (MASCC/ISOO). Systematic review of photobiomodulation for the management of oral mucositis in cancer patients and clinical practice guidelines. Support Care Cancer. 2019 Oct;27(10):3969-3983. doi: 10.1007/s00520-019-04890-2. Epub 2019 Jul 8. PMID: 31286228.
273. Zayed SM, Hakim AAA. Clinical Efficacy of Photobiomodulation on Dental Implant Osseointegration: A Systematic Review. Saudi J Med Med Sci. 2020 May-Aug;8(2):80-86. doi: 10.4103/sjmms.sjmms_410_19. Epub 2020 Apr 17. PMID: 32587488; PMCID: PMC7305678.
274. Zeng J, Wang C, Chai Y, Lei D, Wang Q. Can transcranial photobiomodulation improve cognitive function in TBI patients? A systematic review. Front Psychol. 2024;15:1378570. Published 2024 Jun 17. doi:10.3389/fpsyg.2024.1378570.
275. Zhang W, Hu L, Zhao W, Yan Z. Effectiveness of photobiomodulation in the treatment of primary burning mouth syndrome-a systematic review and meta-analysis. Lasers Med Sci. 2021 Mar;36(2):239-248. doi: 10.1007/s10103-020-03109-9. Epub 2020 Jul 29. PMID: 32725428.
276. Zhang B, Huang X, Huo S, Zhang C, Cen X, Zhao Z. Effect of photobiomodulation therapy on mini-implant stability: a systematic review and meta-analysis. Lasers Med Sci. 2021 Oct;36(8):1557-1566. doi: 10.1007/s10103-021-03281-6. Epub 2021 Mar 4. PMID: 33660109.
277. Zhang J, Zhao Y, Zhao X, Zhang J, Jing L. Efficacy and safety of red and infrared light in the adjunctive treatment on diabetic foot ulcers: A systematic review and meta-analysis. Complement Ther Clin Pract. 2024;57:101906. doi:10.1016/j.ctcp.2024.101906.
278. Zhao H, Hu J, Zhao L. The effect of low-level laser therapy as an adjunct to periodontal surgery in the management of postoperative pain and wound healing: a systematic review and meta-analysis. Lasers Med Sci. 2021 Feb;36(1):175-187. doi: 10.1007/s10103-020-03072-5. Epub 2020 Jul 1. PMID: 32613416.
279. Zheng DH, Hou FC, Zang YJ, Li B. Effects of low-level laser therapy on orthodontic miniscrew stability: a systematic review. Eur J Med Res. 2023 Jan 27;28(1):50. doi: 10.1186/s40001-023-01010-z. PMID: 36707888; PMCID: PMC9881366.
280. Zhi C, Guo Z, Wang T, Liu D, Duan X, Yu X, Zhang C. Viability of Photobiomodulaton Therapy in Decreasing Orthodontic-Related Pain: A Systematic Review and Meta-Analysis. Photobiomodul Photomed Laser Surg. 2021 Aug;39(8):504-517. doi: 10.1089/photob.2021.0035. Epub 2021 Jul 29. PMID: 34328796.
281. Zhou Y, Chia HWA, Tang HWK, Lim SYJ, Toh WY, Lim XL, Cheng LJ, Lau Y. Efficacy of low-level light therapy for improving healing of diabetic foot ulcers: A systematic review and meta-analysis of randomized controlled trials. Wound Repair Regen. 2021 Jan;29(1):34-44. doi: 10.1111/wrr.12871. Epub 2020 Oct 30. PMID: 33078478.
282. Zhu G, Tong Q, Ye X, Li J, Zhou L, Sun P, Liang F, Zhong S, Cheng R, Zhang J. Phototherapy for Cognitive Function in Patients With Dementia: A Systematic Review and Meta-Analysis. Front Aging Neurosci. 2022 Jun 30;14:936489. doi: 10.3389/fnagi.2022.936489. PMID: 35847661; PMCID: PMC9284896.
283. Zhu Z, Zhang R, Chi Y, Li W, Gong W. Photobiomodulation effects on cognitive function - a systematic review and meta-analysis of randomized controlled trials. Lasers Med Sci. 2025;40(1):234. Published 2025 May 21. doi:10.1007/s10103-025-04484-x.
284. Zuccaro J, Ziolkowski N, Fish J. A Systematic Review of the Effectiveness of Laser Therapy for Hypertrophic Burn Scars. Clin Plast Surg. 2017 Oct;44(4):767-779. doi: 10.1016/j.cps.2017.05.008. Epub 2017 Jul 10. PMID: 28888302.
285. Zwiri A, Alrawashdeh MA, Khan M, Ahmad WMAW, Kassim NK, Ahmed Asif J, Suan Phaik K, Husein A, Ab-Ghani Z. Effectiveness of the Laser Application in Temporomandibular Joint Disorder: A Systematic Review of 1172 Patients. Pain Res Manag. 2020 Sep 11;2020:5971032. doi: 10.1155/2020/5971032. PMID: 33005278; PMCID: PMC7503120.
