# Supplementary material for: Adherence to Protocol Registration Among Systematic Reviews on Photobiomodulation: A Meta‐Research Study
Source: J Eval Clin Pract. 2026 Jan 8;32(1):e70346. doi: 10.1111/jep.70346 (PMC12783942; doi:10.1111/jep.70346)
Supplement: Supplementary file 4 — Online Resource 4. [file JEP-32-0-s002.docx]

Supplementary material 4. Characteristics of each included systematic review

|  | **Author, year** | **Type and number of included primary studies** | **Protocol register** | **PROSPERO (or other platforms) register** | **Was the review conducted in full accordance with the registered protocol?** | **Review status on PROSPERO/ other platform** | **Target condition** |
| --- | --- | --- | --- | --- | --- | --- | --- |
|  | Afifi 2017 | RCT/NRSI (n=11) | No |  |  |  | Androgenetic alopecia |
|  | Aguiar 2021 | RCT/NRSI (n=7) | Yes | CRD42020137435 | Yes | Review Ongoing | Radiation dermatitis |
|  | Aguirra 2025 | RCT (n=12) | Yes | CRD42023430324 | Major deviations in databases searched and intervention characteristics | Review Ongoing | Localized muscle resistance |
|  | Ahmad 2020 | RCT (n=37) | No |  |  |  | Temporomandibular joint disorders |
|  | Ahmad 2021 | RCT (n=10) | Yes | CRD42020206228 | Yes | Review Ongoing | Knee osteoarthritis |
|  | Ahmed 2020 | RCT (n=5) | No |  |  |  | Aphthous ulcers |
|  | Akram 2016 | RCT/NRSI (n=18) | Yes | CRD42016043715 | Yes | Review Ongoing | Periodontal disease |
|  | Akram 2017 | RCT/NRSI (n=5) | Yes | CRD42017062401 | The protocol planned to assess non-RCTs using STROBE (a reporting guideline rather than a bias tool), but the published review used the Downs & Black checklist | Review Ongoing | Oral lichen planus |
|  | Akram 2018 | RCT (n=4) | No |  |  |  | Gingival recession defects |
|  | Al-maweri 2016 | RCT/NRSI (n=10) | No |  |  |  | Burning mouth syndrome |
|  | Al-maweri 2017 | RCT (n=6) | No |  |  |  | Oral lichen planus |
|  | Al-maweri 2018 | RCT (n=6) | Yes | CRD42018088526 | Yes | Review Completed | Herpes labialis |
|  | Al-Shahrani 2019 | RCT/NRSI (n=12) | Yes | CRD42019119743 | Yes | Review Ongoing | Orthodontic tooth movement |
|  | Al-Shibani 2018 | RCT (n=4) | No |  |  |  | Gingival recession |
|  | Al-Zainal 2025 | RCT (n=24) | Yes | INPLASY 2024110096 | Yes | Review Ongoing | Diabetic |
|  | Alaql 2025 | RCT (n=11) | Yes | CRD42024591478 | Yes | Review Ongoing | Myopia in children |
|  | Alayat 2024 | RCT (n=6) | Yes | CRD42021292930 | Yes | Review Ongoing | Ankle sprain |
|  | Albaker 2018 | RCT/NRSI (n=5) | No |  |  |  | Peri-implant mucositis |
|  | Allameh 2021 | RCT/NRSI (n=53) | No |  |  |  | Pregnancy |
|  | Alonaizan 2019 | RCT (n=5) | No |  |  |  | Post-operative endodontic pain |
|  | Althuhafy 2024 | RCT (n=13) | Yes | CRD42024504876 | GRADE approach mentioned in the protocol but it's not in the final study | Review Ongoing | Dental injection pain |
|  | Alvarez-Martínez 2025 | RCT/NRSI (n=5) | No |  |  |  | Exercise performance and recovery |
|  | Amiri 2024 | RCT (n=10) | Yes | CRD42024528654 | Yes | Review Ongoing | Bell’s palsy |
|  | Anagnostaki 2020 | RCT (n=17) | No |  |  |  | Endodontic therapy |
|  | Anju 2019 | RCT/NRSI (n=6) | No |  |  |  | Painful diabetic peripheral neuropathy |
|  | Anschau 2019 | RCT (n=5) | Yes | CRD42017077920 | Not all outcomes outlined in the protocol (such as opioid use and parenteral nutrition) were addressed in the published review | Review Ongoing | Cancer therapy-induced oral mucositis |
|  | Austin 2017 | RCT/NRSI (n=27) | No |  |  |  | Malignant melanoma |
|  | Ayen-Rodriguez 2022 | RCT/NRSI (n=20) | Yes | CRD42022313274 | Yes | Review Ongoing | Actinic Cheilitis |
|  | Bai 2014 | RCT/NRSI (n=7) | No |  |  |  | Bladder cancer |
|  | Bakdach 2020 | RCT (n=25) | No |  |  |  | Orthodontic tooth movement |
|  | Baxter 2017 | RCT (n=7) | No |  |  |  | Breast cancer-related lymphedema |
|  | Bekhet 2017 | RCT (n=8) | Yes | CRD42016050283 | Deviation from the planned comparator (placebo) by including splinting-only groups | Review Ongoing | Carpal tunnel syndrome |
|  | Bittencourt 2017 | RCT (n=3) | Yes | CRD42016043258 | Yes | Review Completed | Neurosensory disorders after orthognathic surgery |
|  | Bjordal 2003 | RCT (n=14) | No |  |  |  | Chronic joint disorders |
|  | Bjordal 2007 | RCT (n=36) | No |  |  |  | Knee osteoarthritis pain |
|  | Bjordal 2008 | RCT (n=13) | No |  |  |  | Lateral epicondylitis |
|  | Bjordal 2011 | RCT (n=11) | No |  |  |  | Cancer therapy-induced oral mucositis |
|  | Borsa 2013 | RCT/NRSI (n=10) | No |  |  |  | Exercise performance and recovery |
|  | Brignardello-Petersen 2012 | RCT/NRSI (n=10) | No |  |  |  | Surgical removal of impacted mandibular third molars |
|  | Burger 2017 | RCT (n=9) | No |  |  |  | Carpal tunnel syndrome |
|  | Calarga 2024 | RCT (n=20) | Yes | CRD42023418109 | Yes | Review Completed | Oral mucositis in pediatric patients |
|  | Camolesi 2022 | RCT (n=7) | Yes | CRD42016048914 | The planned risk-of-bias tool (CASP) was replaced with the Jadad scale in the paper without justification | Review Ongoing | Burning mouth syndrome |
|  | Camolesi 2024 | RCT (n=22) | Yes | CRD42023429966 | More databases were used than those specified in the PROSPERO protocol | Review Ongoing | Postoperative complications after mandibular third molar extraction |
|  | Campos 2020 | RCT (n=13) | Yes | CRD42018105658 | Yes | Review Ongoing | Cancer therapy-induced oral mucositis |
|  | Carneiro 2022 | RCT (n=6) | Yes | CRD42020206317 | Yes | Review Ongoing | Tooth sensitivity after dental bleaching |
|  | Cerqueira 2025 | RCT (n=7) | Yes | CRD42023424401 | Yes | Review Ongoing | Hypersensitivity |
|  | Chang 2010 | RCT (n=10) | No |  |  |  | Lateral epicondylitis |
|  | Chaple Gil 2025 | RCT (n=19) | Yes | CRD420251012222 | Yes | Review Ongoing | Oral surgery |
|  | Chen 2019 | RCT (n=9) | Yes | CRD42018102107 | More databases were used than those specified in the PROSPERO protocol | Review Ongoing | Breast cancer-related lymphedema |
|  | Chen 2019(a) | RCT/NRSI (n=8) | No |  |  |  | Dental implant stability |
|  | Chen 2019(b) | RCT (n=7) | No |  |  |  | Root canal treatment |
|  | Chen 2020 | RCT (n=11) | Yes | CRD42020209916 | Yes | Review Ongoing | Tinnitus |
|  | Chen 2021 | RCT/NRSI (n=29) | No |  |  |  | Keloid |
|  | Chen 2024 | RCT/NRSI (n=20) | Yes | CRD42024516676 | Yes | Review Ongoing | Myopia |
|  | Chen 2025 | RCT (n=11) | Yes | CRD42024618702 | More databases were used than those specified in the PROSPERO protocol; exclusion of planned databases (BVS, PEDro, and CINAHL) | Review Ongoing | Diabetic foot ulcer |
|  | Chen 2025(b) | RCT (n=6) | Yes | CRD42024619964 | Yes | Review Ongoing | Age‐Related Macular Degeneration |
|  | Chow 2005 | RCT (n=5) | No |  |  |  | Neck pain |
|  | Clijsen 2017 | RCT/NRSI (n=18) | No |  |  |  | Muskuloesqueletical disorders |
|  | Coluzzi 2020 | RCT (n=20) | No |  |  |  | Non-surgical periodontal therapy |
|  | Costa 2021 | RCT/NRSI (n=6) | Yes | CRD42020188469 | Yes | Review Ongoing | Orthodontic mini-implants stability |
|  | Cronshaw 2019 | RCT/NRSI (n=16) | No |  |  |  | Orthodontic tooth movement |
|  | Cronshaw 2020 | RCT (n=38) | No |  |  |  | Dentistry |
|  | Davoudi 2018 | RCT (n=4) | No |  |  |  | Rapid maxillary expansion |
|  | Davoudi 2018(a) | RCT (n=4) | No |  |  |  | Denture stomatitis |
|  | Dawdy 2017 | RCT (n=21) | No |  |  |  | Surgical removal of impacted mandibular third molars |
|  | da Silva Mira 2024 | RCT/NRSI (n=7) | No |  |  |  | Temporomandibular joint disorders |
|  | de Arruda 2022 | RCT (n=2) | Yes | CRD42020201237 | Yes | Review Ongoing | Tonsillectomy |
|  | de Barros 2022 | RCT (n=15) | Yes | CRD42020155301 | Yes | Review Ongoing | Lower third molar extractions |
|  | de Carvalho 2022 | RCT/NRSI (n=17) | Yes | CRD42020200843 | Yes | Review Ongoing | Gingival lesions resulting from autoimmune diseases |
|  | de Lima 2020 | RCT (n=4) | Yes | CRD42018108380 | Yes | Review Ongoing | Cancer therapy-induced oral mucositis |
|  | de Marchi 2022 | RCT (n=8) | Yes | CRD42020206924 | Yes | Review Completed | Exercise-induced oxidative stress |
|  | de Oliveira 2021 | RCT (n=10) | Yes | CRD42020189290 | Yes | Review Ongoing | Third molar surgery |
|  | de Pedro 2019 | RCT/NRSI (n=13) | No |  |  |  | Neuropathic orofacial pain |
|  | Deana 2017 | RCT (n=20) | No |  |  |  | Orthodontic pain |
|  | Delaney 2017 | RCT (n=5) | No |  |  |  | Androgenic alopecia |
|  | Díaz 2025 | RCT (n=44) | Yes | CRD42025646377 | Yes | Review Completed | Temporomandibular joint disorders |
|  | Díaz 2025 (b) | RCT (n=18) | Yes | CRD42025630135 | Yes | Review Ongoing | Maxillofacial neuropathies |
|  | dos Santos 2019 | RCT (n=9) | Yes | CRD42017078269 | Yes | Review Completed | Maxillofacial trauma |
|  | dos Santos 2019(a) | RCT (n=7) | Yes | CRD42018094017 | Yes | Review Ongoing | Plantar fasciitis |
|  | Ebrahimi 2021 | RCT (n=12) | Yes | CRD42020192403 | Yes | Review Completed | Gingival wound healing |
|  | Elmsmari 2024 | RCT (n=17) | Yes | CRD42023415417 | Yes | Review Ongoing | Pain management after endodontic treatment |
|  | Escudero 2019 | RCT/NRSI (n=37) | No |  |  |  | Bone repair |
|  | Ezzati 2020 | RCT (n=19) | No |  |  |  | Musculoskeletal pain |
|  | Farzan 2021 | RCT (n=4) | No |  |  |  | Pain induced by orthodontic separator placement |
|  | Farzan 2022 | RCT (n=4) | No |  |  |  | Bone formation in rapid palatal expansion |
|  | Ferrillo 2022 | RCT (n=16) | Yes | CRD42021251904 | Yes | Review Ongoing | Temporomandibular joint disorders |
|  | Figueiredo 2013 | NRSI (n=12) | No |  |  |  | Oral mucositis |
|  | Firoozi 2020 | RCT (n=8) | Yes | CRD42020205952 | Yes | Review Completed | Recovery from neurosensory disturbance after sagittal split ramus osteotomy |
|  | Flemming 1999 | RCT (n=4) | No |  |  |  | Venous leg ulcers |
|  | Fornaini 2019 | RCT/NRSI (n=19) | No |  |  |  | Pediatric dentistry |
|  | Franke 2017 | RCT (n=17) | No |  |  |  | Carpal tunnel syndrome |
|  | Gaitero 2025 | RCT (n=3) | Yes | CRD42023403779 | Yes | Review Ongoing | Nipple trauma and pain during breastfeeding |
|  | Galiano-Castillo 2020 | RCT (n=11) | Yes | CRD42020151145 | Yes | Review Completed | Xerostomia |
|  | Garola 2021 | RCT/NRSI (n=17) | No |  |  |  | Alveolar osteitis |
|  | Gavish 2019 | RCT/NRSI (n=11) | Yes | CRD42018091415 | Yes | Review Completed | Home-use photobiomodulation devices |
|  | Ge 2014 | RCT/NRSI (n=9) | No |  |  |  | Accelerated orthodontic tooth movement |
|  | Gkantidis 2014 | RCT/NRSI (n=18) | No |  |  |  | Accelerated orthodontic tooth movement |
|  | Godaert 2024 | RCT/NRSI (n=10) | Yes | CRD42024504081 | Yes | Review Ongoing | Older adults (≥65 years) with diverse conditions |
|  | Golež 2021 | RCT/NRSI (n=18) | No |  |  |  | Xerostomia related to hyposalivation |
|  | Gomes 2022 | RCT (n=4) | Yes | CRD42021223429 | Yes | Review Ongoing | Headache |
|  | Gondivkar 2020 | RCT/NRSI (n=7) | No |  |  |  | Oral submucous fibrosis |
|  | Gross 2007 | RCT (n=88) | No |  |  |  | Mechanical neck disorders |
|  | Gross 2013 | RCT (n=17) | No |  |  |  | Neck Pain |
|  | Guimarães 2022 | RCT (n=14) | No |  |  |  | Plantar fasciitis |
|  | Gupta 2020 | RCT (n=15) | No |  |  |  | Androgenetic alopecia |
|  | Gutiérrez-Menéndez 2020 | RCT/NRSI (n=26) | No |  |  |  | Psychological disorders |
|  | Hadis 2016 | RCT/NRSI (n=56) | No |  |  |  | Radiometry |
|  | Haghighat 2024 | RCT (n=13) | No |  |  |  | Trigeminal Neuralgia |
|  | Hakimiha 2021 | RCT/NRSI (n=7) | Yes | CRD42020163799 | More outcomes were reported in the paper compared to the protocol. | Review Completed | Inferior alveolar nerve injury following oral surgical procedures |
|  | Han 2016 | RCT (n=10) | No |  |  |  | Aphthous stomatitis |
|  | Hanna 2021 | RCT (n=44) | Yes | CRD42020198921 | More databases were used than those specified in the PROSPERO protocol, and some additional topics were included in the assessment (two reviews shared the same protocol). | Review Ongoing | Temporomandibular joint disorders |
|  | Hanna 2021(a) | RCT (n=12) | Yes | CRD42020198921 | More databases were used than those specified in the PROSPERO protocol, and some additional topics were included in the assessment (two reviews shared the same protocol). | Review Ongoing | Burning mouth syndrome |
|  | Haslerud 2014 | RCT (n=17) | No |  |  |  | Shoulder tendinopathy |
|  | He 2012 | RCT/NRSI (n=5) | No |  |  |  | Orthodontic pain |
|  | He 2014 | RCT (n=12) | No |  |  |  | Mandibular third molar surgery |
|  | He 2017 | RCT (n=8) | No |  |  |  | Chemotherapy-induced oral mucositis |
|  | Heiskanen 2020 | RCT (n=5) | No |  |  |  | Cancer treatment-related salivary gland dysfunction |
|  | Herpich 2014 | RCT (n=11) | No |  |  |  | Temporomandibular joint disorders |
|  | Hosseinpour 2019 (a) | RCT/NRSI (n=20) | No |  |  |  | Bone regeneration |
|  | Hosseinpour 2019 (b) | RCT/NRSI (n=46) | No |  |  |  | Oral surgery |
|  | Huang 2015 | RCT (n=9) | No |  |  |  | Knee osteoarthritis |
|  | Huang 2015(b) | RCT (n=7) | No |  |  |  | Chronic low back pain |
|  | Huang 2023 | RCT/NRSI (n=8) | Yes | CRD42022355857 | Yes | Review Ongoing | Orthodontic tooth movement |
|  | Huisstede 2017 | RCT/NRSI (n=24) | No |  |  |  | Carpal tunnel syndrome |
|  | Ibarra 2020 | RCT/NRSI (n=6) | Yes | CRD42020181289 | Yes | Review Completed | Trigeminal neuralgia |
|  | Imani 2018 | RCT (n=6) | No |  |  |  | Orthodontic tooth movement |
|  | Jajarm 2018 | RCT/NRSI (n=15) | No |  |  |  | Oral lichen planus |
|  | Jang 2012 | RCT (n=22) | No |  |  |  | Joint pain relief |
|  | Javaherian 2020 | RCT (n=4) | No |  |  |  | Bell's palsy |
|  | Jedliński 2020 | RCT (n=8) | No |  |  |  | Orthodontic tooth movement |
|  | Ji 2023 | RCT (n=11) | Yes | CRD42023444677 | More databases were used than those specified in the PROSPERO protocol | Review Ongoing | Depression |
|  | Jiménez 2024 | RCT/NRSI (n=8) | No |  |  |  | Spastic cerebral palsy in children |
|  | John 2016 | RCT/NRSI (n=22) | No |  |  |  | Hidradenitis suppurativa |
|  | Joseph 2025 | RCT/NRSI (n=5) | Yes | CRD42024533192 | Yes | Review Ongoing | Cancer therapy-induced oral mucositis |
|  | Kadhim-Saleh 2013 | RCT (n=8) | No |  |  |  | Neck pain |
|  | Karlsson 2008 | RCT (n=4) | No |  |  |  | Chronic periodontitis |
|  | Kauark-Fontes 2021 | RCT/NRSI (n=4) | Yes | CRD42019133695 | Yes | Review Completed | Cancer treatment toxicities |
|  | Kechichian 2020 | RCT/NRSI (n=17) | No |  |  |  | Keratosis pilaris |
|  | Khalil 2024 | RCT/NRSI (n=4) | Yes | CRD42023441214 | More databases were used than those specified in the PROSPERO protocol | Review Ongoing | Cancer therapy-induced oral mucositis |
|  | Khan 2025 | RCT/NRSI (N=14) | Yes | CRD42024567428 | Yes | Review Completed | Pulpotomy of primary teeth |
|  | Khemiss 2024 | RCT (n=9) | Yes | CRD42023402297 | Yes | Review Ongoing | Burning mouth syndrome |
|  | Lai 2022 | RCT (n=22) | Yes | CRD42021260954 | More outcomes were reported in the paper compared to the protocol. | Review Completed | Melasma |
|  | Lauxen 2025 | RCT (n=13) | Yes | https://doi.  org/10.17605/OSF.IO/HQCRP |  | Not reported | Carpal tunnel syndrome |
|  | Leal-Junior 2015 | RCT (n=13) | No |  |  |  | Exercise performance and recovery |
|  | Li 2015 | RCT (n=11) | No |  |  |  | Orthodontic pain |
|  | Li 2016 | RCT (n=7) | No |  |  |  | Carpal tunnel syndrome |
|  | Li 2018 | RCT (n=7) | No |  |  |  | Diabetic foot ulcer |
|  | Lima 2012 | NRSI (n=4) | No |  |  |  | Breast cancer-related lymphedema |
|  | Lin 2024 | RCT/NRSI (n=12) | Yes | CRD42023414640 | Yes | Review Ongoing | Bell’s palsy |
|  | Lin 2025 | RCT/NRSI (n=8) | Yes | CRD42024576164 | More databases were used than those specified in the PROSPERO protocol | Review Ongoing | Acute radiation dermatitis |
|  | Liu 2019 | RCT (n=8) | No |  |  |  | Androgenic alopecia |
|  | Louzeiro 2020 | NRSI (n=6) | Yes | CRD42019139620 | Yes | Review Ongoing | Prevent hyposalivation in patients undergoing head and neck radiotherapy |
|  | Lu 2025 | RCT (n=12) | Yes | CRD42023384852 | Yes | Review Ongoing | Oral lichen planus |
|  | Luo 2022 | RCT (n=24) | Yes | CRD42020200740 | Yes | Review Ongoing | Muscular performance and soreness recovery in athletes |
|  | Luo 2024 | RCT (n=22) | Yes | CRD42023402872 | Yes | Review Completed | Post-operative endodontic pain |
|  | Machado 2017 (a) | RCT/NRSI (n=3) | No |  |  |  | Dentin hypersensitivity |
|  | Machado 2017 (b) | RCT/NRSI (n=4) | Yes | CRD42016036648 | Yes | Review Completed | Pressure ulcers |
|  | Machado 2018 | RCT (n=14) | Yes | CRD42017056877 | Yes | Review Ongoing | Management of creatine kinase activity in general versus localized exercise |
|  | Mahintach 2024 | RCT/NRSI (n=12) | Yes | CRD42023467581 | Yes | Review Ongoing | Alveolar ridge preservation after dental extraction |
|  | Mahmood 2022 | RCT/NRSI (n=8) | Yes | CRD42022315076 | Yes | Review Completed | Breast cancer-related lymphedema |
|  | Mahuli 2024 | RCT (n=10) | Yes | CRD42023394321 | Yes | Review Ongoing | Oral lichen planus |
|  | Maia 2012 | RCT (n=14) | No |  |  |  | Temporomandibular joint disorders |
|  | Malik 2023 | RCT (n=14) | Yes | CRD42021225106 | Yes | Review Ongoing | Knee osteoarthritis |
|  | Martimbianco 2020 | NRSI (n=4) | Yes | CRD42019123185 | Yes | Review Ongoing | Achilles tendinopathy |
|  | Matos 2021 | RCT/NRSI (n=8) | Yes | CRD42021226064 | Yes | Review Completed | Burning oral syndrome |
|  | Máximo 2022 | RCT (n=6) | Yes | CRD42020187091 | While the protocol focused on PBM for masticatory or mandibular function in adults aged 18–60, the review targeted burning mouth syndrome with no age, language, or date restrictions. | Review Ongoing | Temporomandibular joint disorders |
|  | Melis 2012 | RCT (n=14) | No |  |  |  | Temporomandibular joint disorders |
|  | Meneses-Santos 2022 | RCT (n=5) | Yes | CRD42020156786 | GRADE approach mentioned in the protocol, but it's not in the final study | Review Ongoing | Orthognathic surgery |
|  | Mesquita 2024 | RCT/NRSI (n=8) | Yes | CRD42022304740 | Yes | Review Completed | Anesthetic puncture of dental local anesthesia |
|  | Michelogiannakis 2022 | RCT (n=6) | Yes | CRD42021230291 | Yes | Review Ongoing | Stability of orthodontic mini-screw implants |
|  | Migliorati 2013 | RCT/NRSI (n=24) | No |  |  |  | Oral mucositis in cancer patients |
|  | Mikami 2020 | RCT (n=10) | No |  |  |  | Non-surgical and surgical periodontal therapy |
|  | Minervini 2024 | RCT/NRSI (n=5) | Yes | CRD4453468737 | Protocol not found in the PROSPERO platform | Not found | Dry socket pain |
|  | Mirzaei 2019 | RCT (n=7) | No |  |  |  | Inferior alveolar nerve damage after sagittal split osteotomy |
|  | Mokeem 2018 | RCT/NRSI (n=4) | No |  |  |  | Aggressive periodontitis |
|  | Munguia 2018 | RCT/NRSI (n=8) | No |  |  |  | Temporomandibular myofascial pain |
|  | Najem 2018 | RCT/NRSI (n=11) | No |  |  |  | Androgenic alopecia |
|  | Nampo 2016 (a) | RCT (n=15) | No |  |  |  | Exercise capacity and muscle performance |
|  | Nampo 2016 (b) | RCT (n=13) | No |  |  |  | Delayed onset muscle soreness |
|  | Naterstad 2022 | RCT (n=18) | Yes | CRD42017077511 | Some planned outcomes (QoL and global impression) were not reported in the review publication | Review Ongoing | Lower extremity tendinopathy or plantar fasciitis |
|  | Navarro-Fernández 2023 | RCT (n=5) | Yes | CRD42021254655 | Yes | Review Ongoing | Orthognathic surgery |
|  | Nayyer 2022 | RCT (n=6) | Yes | CRD42020167291 | Yes | Review Ongoing | Orthodontic tooth movement |
|  | Nogueira 2015 | RCT (n=3) | No |  |  |  | Tendinopathy |
|  | Nunes 2024 | RCT (n=5) | Yes | CRD42021243500 | Yes | Review Ongoing | Pain in endodontic reintervention |
|  | Oberoi 2014 | RCT (n=18) | No |  |  |  | Oral mucositis |
|  | Okuhara 2025 | RCT (n=7) | Yes | CRD42019119287 | Yes | Review Ongoing | Burning Mouth Syndrome |
|  | Oliveira 2024 | RCT (n=10) | Yes | CRD42022338150 | Yes | Review Ongoing | Knee osteoarthritis |
|  | Olszewska 2025 | RCT/NRSI (n=14) | Yes | <https://doi.org/10.17605/OSF.IO/6XJ7T> | Yes | Not reported | Neurosensory disturbances in orthognathic surgery |
|  | Omar 2012 | NRSI (n=8) | No |  |  |  | Breast cancer-related lymphedema |
|  | Pacheco 2022 | NRSI (n=5) | Yes | CRD42021250106 | Yes | Review Completed | Oral manifestations of patients  infected by Sars‐CoV‐2 |
|  | Paglioni 2019 | RCT/NRSI (n=15) | Yes | CRD42018115503 | Yes | Review Completed | Pain related to head and neck cancer treatment |
|  | Parker 2019 | RCT/NRSI (n=52) | No |  |  |  | Dental (clinical dentistry) |
|  | Parker 2020 | RCT (n=25) | No |  |  |  | Oral soft tissue outcomes |
|  | Parra-Rojas 2025 | RCT (n=13) | Yes | CRD42023465329 | GRADE approach mentioned in the protocol but it's not in the final study | Review Ongoing | Chemotherapy-induced oral mucositis |
|  | Pavlic 2012 | NRSI (n=12) | No |  |  |  | Xerostomia |
|  | Penberthy 2021 | RCT/NRSI (n=22) | No |  |  |  | Various medical indications |
|  | Peng 2020 | RCT (n=30) | No |  |  |  | Oral mucositis |
|  | Peralta-Mamani 2019 | NRSI (n=7) | Yes | CDR42017080523 | Protocol not found in the PROSPERO platform | Not found | Radiotherapy-induced Oral Mucositis |
|  | Petrucci 2011 | RCT (n=6) | No |  |  |  | Temporomandibular joint disorders |
|  | Petz 2020 | RCT (n=5) | No |  |  |  | Pressure ulcers |
|  | Qadri 2015 | NRSI (n=10) | No |  |  |  | Chronic periodontitis |
|  | Radithia 2024 | RCT (n=14) | Yes | CRD42022355737 | Yes | Review Ongoing | Recurrent aphthous stomatitis |
|  | Rajai Firouzabadi 2024 | RCT/NRSI (n=16) | Yes | CRD42023487916 | Yes | Review Completed | Allergic rhinitis |
|  | Ramezani 2020 | NRSI (n=16) | No |  |  |  | Spinal cord injury |
|  | Rani 2025 | RCT (n=15) | Yes | CRD42024515699 | More databases were used than those specified in the PROSPERO protocol. The published study narrowed the eligibility to include only RCTs, diverging from the broader range of study designs initially planned. | Review Completed | Dental implant stability |
|  | Rayegani 2017 | RCT (n=14) | No |  |  |  | Knee osteoarthritis |
|  | Rayegani 2019 | RCT (n=6) | No |  |  |  | Carpal tunnel syndrome |
|  | Redman 2022 | RCT (n=5) | Yes | CRD42018099772 | Yes | Review Ongoing | Oral mucositis in children with cancer |
|  | Reis 2022 | RCT (n=19) | No |  |  |  | Regeneration in tissue |
|  | Ren 2015 | RCT (n=14) | No |  |  |  | Orthodontic pain |
|  | Ren 2016 | RCT (n=8) | No |  |  |  | Non-surgical periodontal treatment |
|  | Ribeiro 2024 | RCT/NRSI (n=4) | Yes | CRD42023433241 | Yes | Review Completed | Xerostomia patients undergoing head and neck radiotherapy |
|  | Ricci 2010 | NRSI (n=7) | No |  |  |  | Fibromyalgia syndrome |
|  | Rocha 2022 | RCT (n=4) | Yes | CRD42021231565 | Yes | Review Ongoing | Radiodermatitis in breast cancer |
|  | Ruiz 2022 | RCT/NRSI (n=7) | Yes | CRD42019154002 | Yes | Review Ongoing | Oral lichen planus |
|  | Sadeghian 2025 | RCT (n=13) | Yes | CRD42024555150 | More databases used than originally planned. | Review Ongoing | Bone regeneration |
|  | Saini 2024 | RCT/NRSI (n=26) | No |  |  |  | Osseointegration in dental implants |
|  | Salehpour 2019 | NRSI (n=9) | No |  |  |  | Cognitive performance |
|  | Saneja 2020 | RCT (n=11) | No |  |  |  | Peri‐implant mucositis |
|  | Salajegheh 2024 | RCT (n=13) | No |  |  |  | Rheumatoid arthritis |
|  | Sales 2025 | RCT/NRSI (n=7) | Yes | CRD42023468625 | Yes | Review Ongoing | Chemo/radiotherapy-induced oral mucositis |
|  | Santinoni 2017 | NRSI (n=15) | Yes | CRD42016041899 | Yes | Review Ongoing | Bone maxillofacial defects |
|  | Santos 2020 | RCT (n=13) | Yes | CRD42019132297 | Yes | Review Completed | Diabetic foot ulcers |
|  | Seyyedi 2024 | RCT (n=9) | No |  |  |  | Postoperative endodontic pain |
|  | Shadid 2014 | NRSI (n=8) | No |  |  |  | Dental implants |
|  | Shafaee 2020 | NRSI (n=5) | No |  |  |  | Alveolar osteitis |
|  | Shan 2021 | RCT/NRSI (n=35) | Yes | CRD42020162721 | Yes | Review Ongoing | Dentin hypersensitivity |
|  | Shukla 2016 | RCT (n=13) | No |  |  |  | Temporomandibular joint disorders |
|  | Sims 2014 | RCT (n=58) | No |  |  |  | Lateral epicondylitis |
|  | Slot 2014 | RCT (n=9) | No |  |  |  | Non-surgical periodontal therapy |
|  | Smoot 2015 | RCT/NRSI (n=9) | No |  |  |  | Breast cancer-related lymphedema |
|  | Sobol 2024 | RCT/NRSI (n=10) | No |  |  |  | Myopia |
|  | Sobral 2021 | RCT (n=17) | Yes | CRD42019131016 | Yes | Review Ongoing | Myofascial temporomandibular disorder |
|  | Soh 2024 | RCT/NRSI (n=8) | Yes | CRD42023428626 | Yes | Review Ongoing | Oral lichen planus |
|  | Stausholm 2019 | NRSI (n=22) | Yes | CRD42016035587 | Yes | Review Completed | Knee osteoarthritis |
|  | Sun 2024 | RCT/NRSI (n=9) | Yes | CRD42024475312 | Yes | Review Ongoing | Cleft lip or/and palate scarring |
|  | Sun 2025 | RCT (n=11) | Yes | CRD42024532988 | Yes | Review Ongoing | Health indicators in obese patients |
|  | Sussmilch-Leitch 2012 | RCT (n=19) | No |  |  |  | Achilles tendinopathy |
|  | Taberner-Vallverdú 2015 | NRSI (n=8) | No |  |  |  | Dry socket |
|  | Taha 2024 | RCT (n=18) | No |  |  |  | Skin Wounds |
|  | Talluri 2022 | RCT (n=10) | No |  |  |  | Tinnitus |
|  | Tan 2024 | RCT/NRSI (n=6) | Yes | PCOS guideline technical report.20 |  | Not reported | Hirsutism in women with polycystic ovarian syndrome |
|  | Tchande-Fossuo 2016 | RCT (n=4) | Yes | CRD42015029825 | Yes | Review Ongoing | Diabetic foot ulcer |
|  | Tehrani 2022 | RCT (n=13) | No |  |  |  | Myofascial neck pain syndrom |
|  | Telles-Araujo 2024 | RCT (n=10) | Yes | CRD42024503264 | More databases were used than those specified in the PROSPERO protocol | Review Ongoing | Olfactory and gustatory dysfunction post-COVID-19 |
|  | Tengrungsun 2012 | RCT/NRSI (n=33) | No |  |  |  | Orofacial pain |
|  | Tomazoni 2020 | RCT (n=12) | No |  |  |  | Non-specific low back pain |
|  | Toopalle 2024 | RCT(n=3) | No |  |  |  | Postoperative pain and endodontic retreatment |
|  | Tournavitis 2022 | RCT(n=28) | Yes | CRD42021252489 | Yes | Review Ongoing | Temporomandibular joint disorders |
|  | Tripodi 2021 | NRSI (n=17) | Yes | CRD42020202508 | Yes | Review Completed | Tendinopathy |
|  | Tumilty 2010 | RCT/NRSI (n=11) | No |  |  |  | Tendinopathy |
|  | Tuner 2019 | NRSI (n=39) | No |  |  |  | Temporomandibular joint disorders |
|  | Ullah 2025 | RCT (n=10) | No |  |  |  | Myopia |
|  | Vale 2015 | RCT (n=2) | No |  |  |  | Aphthous ulcers |
|  | Vande 2022 | RCT/NRSI (n=7) | Yes | CRD42021249393 | GRADE assessment is mentioned in protocol and not presented in the final publication | Review Ongoing | Dental implants |
|  | Vanin 2017 | NRSI (n=39) | Yes | CRD42015024010 | Yes | Review Ongoing | Muscular performance |
|  | Vieceli 2022 | NRSI (n=6) | Yes | CRD42020178792 | Yes | Review Ongoing | Pressure injuries |
|  | Vlassov 2006 | RCT (n=1) | No |  |  |  | Tuberculosis |
|  | Vrijman 2011 | RCT (n=13) | No |  |  |  | Hypertrophic scars |
|  | Wang 2019 | RCT (n=6) | No |  |  |  | Plantar fasciitis |
|  | Weber 2016 | NRSI (n=10) | No |  |  |  | Osteonecrosis of the jaw |
|  | Wei 2025 | RCT/NRSI (n=9) | Yes | CRD42023482170 | Yes | Review Completed | Upper lip scars in cleft lip patients |
|  | Winters 2013 | RCT (n=11) | No |  |  |  | Tibial stress syndrome |
|  | Wu 2021 | RCT (n=8) | No |  |  |  | Temporomandibular joint disorders |
|  | Xiaoting 2010 | RCT (n=26) | No |  |  |  | Pain during fixed orthodontic appliance |
|  | Xu 2018 | RCT (n=31) | No |  |  |  | Temporomandibular joint disorders |
|  | Yadav 2025 | RCT (n=7) | Yes | CRD42023453098 | Yes | Review Completed | Plantar fasciitis |
|  | Yavagal 2021 | RCT (n=9) | Yes | CRD42019121465 | Yes | Review Ongoing | Orthodontic tooth movement |
|  | Ye 2025 | RCT (n=17) | Yes | CRD42024519830 | Yes | Review Ongoing | Pain perception in local anaesthesia infiltration |
|  | Yeh 2019 | RCT (n=9) | Yes | CRD42017079531 | Yes | Review Completed | Fibromyalgia |
|  | Youssef 2024 | RCT (n=5) | Yes | CRD42023410702 | Cochrane RoB 2 tool was planned; NIH tool was used instead. | Review Ongoing | Myopia |
|  | Zadik 2019 | RCT/NRSI (n=33) | No |  |  |  | Oral mucositis |
|  | Zayed 2020 | RCT/NRSI (n=7) | No |  |  |  | Dental Implant |
|  | Zeng 2024 | NRSI (n=6) | No |  |  |  | Cognitive function in TBI patients |
|  | Zhang 2020 | RCT (n=12) | No |  |  |  | Burning mouth syndrome |
|  | Zhang 2021 | RCT/NRSI (n=7) | Yes | CRD42020182953 | Grey literature databases were not included as planned. “Success rate” added post hoc as secondary outcome | Review Ongoing | Mini-implant stability |
|  | Zhang 2024 | RCT (n=28) | Yes | CRD42023491993 | Yes | Review Ongoing | Diabetic foot ulcers |
|  | Zhao 2021 | RCT (n=13) | No |  |  |  | Periodontal surgery |
|  | Zheng 2023 | RCT (n=3) | No |  |  |  | Orthodontic miniscrew stability |
|  | Zhi 2021 | RCT (n=25) | Yes | CRD42020175620 | Yes | Review Ongoing | Orthodontic-related pain |
|  | Zhou 2021 | RCT (n=12) | Yes | CRD42020187595 | Yes | Review Completed | Diabetic foot ulcers |
|  | Zhu 2022 | RCT/NRSI (n=13) | Yes | CRD42021267596 | Included 10 cognitive scales in the protocol, and 2 in the final study | Review Completed | Dementia |
|  | Zhu 2025 | RCT (n=24) | Yes | CRD42024552832 | Yes | Review Ongoing | Cognitive function |
|  | Zuccaro 2017 | NRSI (n=12) | No |  |  |  | Hypertrophic burn scars |
|  | Zwiri 2020 | RCT/NRSI (n=32) | Yes | CRD42020177562 | Yes | Review Completed | Temporomandibular joint disorders |

**N**: number of included studies, **NIH**: National Institutes of Health, **NRSI**: non-randomized studies of intervention, **PCOS**: International Evidence-Based Guideline for the Assessment and Management of Polycystic Ovary Syndrome, **QoL**: quality of life, **RCT**: randomized clinical trial, **RoB**: risk of bias.
